# Ca Cations Impact the Local Environment inside HZSM-5 Pores during the Methanol-to-Hydrocarbons Reaction

**DOI:** 10.1021/acscatal.3c00059

**Published:** 2023-02-23

**Authors:** Anna Liutkova, Hao Zhang, Jérôme F. M. Simons, Brahim Mezari, Marta Mirolo, Gustavo A. Garcia, Emiel J. M. Hensen, Nikolay Kosinov

**Affiliations:** † Laboratory of Inorganic Materials and Catalysis, Department of Chemical Engineering and Chemistry, Eindhoven University of Technology, P.O. Box 513, 5600 MB Eindhoven, The Netherlands; ‡ ESRF, The European Synchrotron, 71 Avenue des Martyrs, CS40220, 38043 Grenoble, Cedex 9, France; § Synchrotron SOLEIL, L’Orme des Merisiers, St Aubin, B.P. 48, 91192 Gif sur Yvette, France

**Keywords:** methanol-to-hydrocarbons, step-response kinetic experiments, operando spectroscopy, hydrocarbon pool, confinement, zeolite, Ca/ZSM-5

## Abstract

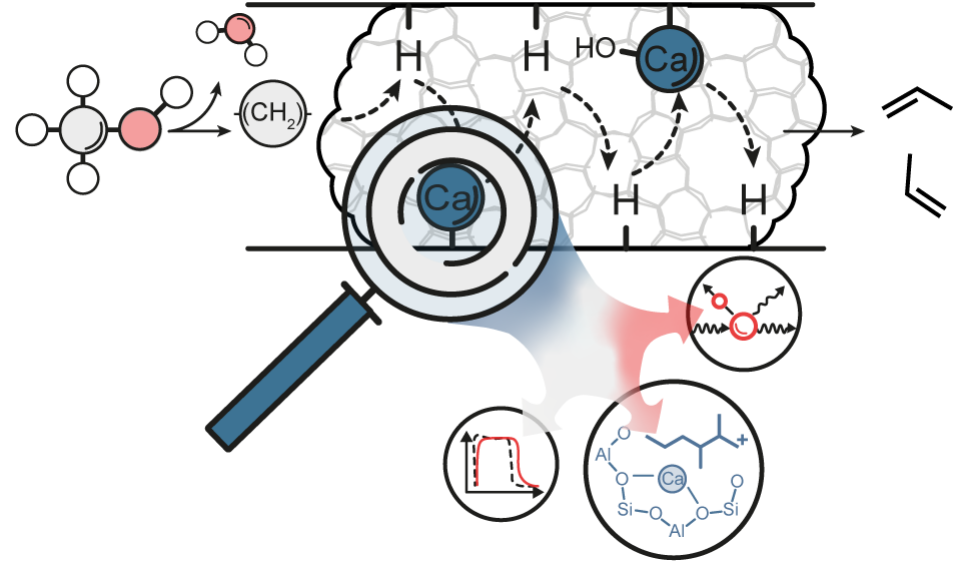

The methanol-to-hydrocarbons (MTH) process is an industrially relevant method to produce valuable light olefins such as propylene. One of the ways to enhance propylene selectivity is to modify zeolite catalysts with alkaline earth cations. The underlying mechanistic aspects of this type of promotion are not well understood. Here, we study the interaction of Ca^2+^ with reaction intermediates and products formed during the MTH reaction. Using transient kinetic and spectroscopic tools, we find strong indications that the selectivity differences between Ca/ZSM-5 and HZSM-5 are related to the different local environment inside the pores due to the presence of Ca^2+^. In particular, Ca/ZSM-5 strongly retains water, hydrocarbons, and oxygenates, which occupy as much as 10% of the micropores during the ongoing MTH reaction. This change in the effective pore geometry affects the formation of hydrocarbon pool components and in this way directs the MTH reaction toward the olefin cycle.

## Introduction

The chemical industry faces a rising demand for light olefins, in particular, propylene.^1^ Currently, propylene is mainly produced from steam cracking of naphtha and gas oil and obtained as a byproduct in oil refineries.^2^ Among alternative technologies such as propane dehydrogenation^3^ and olefin metathesis,^4^ methanol-to-propylene (MTP) is promising, because it can potentially use methanol from renewable sources (e.g., CO_2_ or biomass).^5^ Typical MTP catalysts are based on ZSM-5 zeolite.^6^ MTP technology faces several challenges related to the selectivity and stability of the ZSM-5 catalysts.^7^ Postsynthetic modification of zeolites with metal species has been employed to tune the reaction selectivity.^8^ In particular, modification of ZSM-5 zeolite with alkaline earth cations is an effective way to increase the propylene selectivity.^9^ Recently, Gascon and co-workers investigated in detail such alkaline earth cation modification, providing mechanistic insights into the substantial improvement of the stability and propylene selectivity of ZSM-5.^10−13^ The authors ascribed the better performance to the isolation of Brønsted acid sites and the Lewis acid properties of Ca^2+^ ions. They argued that the alkaline earth modification can lead to destabilization of aromatic intermediates within the dual-cycle hydrocarbon pool mechanism, increasing the selectivity to products of the olefinic cycle in methanol-to-hydrocarbons chemistry.^14^

One aspect that remains underexplored in previous studies is the influence of introducing alkaline earth cations on the local pore environment during conversion of methanol over microporous zeolite. In a previous work, we used a transient pulse reaction technique to show that HZSM-5 and Ca/ZSM-5 contain different amounts of different hydrocarbon pool intermediates. While HZSM-5 mainly contained aromatic hydrocarbons, aliphatics were predominant in the pores of Ca/ZSM-5.^15^ In addition to hydrocarbons, water is another MTH product ofthat might affect the reaction.^16,17^ Recent reports by the group of Lercher emphasize that tighter confinement in zeolite pores, induced by adsorbed water clusters, strongly influences alcohol dehydration activity.^18−20^ Inspired by these findings, we hypothesize that the particular catalytic properties of alkaline earth-modified zeolites can be linked to the modification of the microporous space by strongly adsorbed molecules. In this work we used transient kinetic experiments and characterization techniques, such as nuclear magnetic resonance (NMR) spectroscopy, infrared (IR) spectroscopy, photoelectron-photoion coincidence (PEPICO) spectroscopy, X-ray powder diffraction (XRD) and thermogravimetric (TG) analysis, to study the local pore environment in working HZSM-5 and Ca/ZSM-5 catalysts. Strong adsorption of water, methanol, and hydrocarbons on Ca ions leads to a significantly higher pore occupancy, which can be related to distinct differences in the chemical composition of the hydrocarbon pool components. The present findings help to rationalize the impact of Ca cations on the propylene selectivity and stability of Ca/ZSM-5.

## Experimental Methods

The proton form HZSM-5 was obtained by calcining a commercial NH_4_ZSM-5 powder (Alfa Aesar) in air at 550 °C for 5 h. Ca/ZSM-5 was prepared by impregnation of the HZSM-5 with aqueous solution of Ca(NO_3_)_2_ (Alfa Aesar, 99.0%), aiming at 1 wt % of metal loading. Na/ZSM-5 was prepared by partial ion exchange of the HZSM-5 zeolite in aqueous solution of sodium nitrate NaNO_3_ (≥99% Merck). We aimed to replace a comparable number of Bronsted acid sites (BAS) by ion exchange with Na, as in the Ca/ZSM-5 sample. The most important physicochemical properties of the studied HZSM-5, Ca/ZSM-5 and Na/ZSM-5 catalysts are provided in Table 1. The micropore volumes of the three obtained samples are close to 0.12 cm^3^·g^–1^. The concentration of BAS as probed by ^1^H NMR spectroscopy decreases in the order HZSM-5 ≫ Na/ZSM-5 > Ca/ZSM-5. Modification of ZSM-5 with Na and Ca cations leads to an increased number of Lewis acid sites (LAS) as probed by IR with pyridine. The M/Al atomic ratios of the metal-modified HZSM-5 zeolites were 0.54 for M = Ca and 0.39 for M = Na.

**Table 1:** Physico-Chemical Properties of Zeolite Catalysts

Catalyst	*S*_total_, m^2^·g^–1^	*S*_micro_, m^2^·g^–1^	*S*_external_, m^2^·g^-1^	*V*_micro_, cm^3^·g^-1^	Si/Al^a^	FAl^b^, %	BAS,μmol·g^–1^, ^1^H NMR	M/Al ratio^a^	Exchange degree^c^, %	BAS/LAS, μmol·g^–1^^d^
HZSM-5	364	336	29	0.12	37	93.5	404	n.a.	0	437/85
Ca/ZSM-5	272	243	29	0.11	34	96.7	215	0.54	47	189/361
Na/ZSM-5	291	264	27	0.12	36	93.4	264	0.39	35	268/226

a Measured by ICP elemental analysis.

b Fraction of framework Al as determined by ^27^Al MAS NMR.

c Determined by the fractional occupation of initial BAS by the metal ions as probed by ^1^H NMR spectroscopy.

d IR spectroscopy of adsorbed pyridine.

Further details about the methods of catalyst preparation and basic characterization are provided in the SI.

### Catalytic Activity Measurements

MTH activity tests were performed in a fixed-bed reactor. In a typical experiment, a quartz reactor was charged with 25 mg of the sieved catalyst (250–500 μm pellet size) held between two quartz wool plugs. The catalyst was subsequently pretreated under oxygen atmosphere (20 vol % O_2_ in He) at 550 °C (ramp rate 10 °C·min^–1^) for 1 h to remove organic contamination. After pretreatment, the temperature was set to 350 °C, 400 °C, or 450 °C in pure He. The experiments were carried out in a flow of He, containing 0.75 kPa of methanol in 10 mL·min^–1^ flow at weight hourly space velocity (WHSV) 0.3 h^–1^ for 350 °C; 6 kPa of methanol in 30 mL·min^–1^ for 400 °C (WHSH 6 h^–1^), and 12 kPa of methanol in 30 mL·min^–1^ for 450 °C (WHSV 12 h^–1^), respectively. The reactor outlet was connected to a gas chromatograph (Compact GC 4.0, Global Analyzer Solutions) and a mass spectrometer (Pfeiffer Omnistar) with heated gas transfer lines. The GC was equipped with two precolumns, three columns, and three detectors. A thermal conductivity detector (TCD) coupled with an RT-Q-Bond precolumn (length 3 m; i.d. 0.32 mm; film thickness 10 μm) and a Molsieve 5A FS column (Restek, length 10 m; i.d. 0.32 mm; film thickness 30 μm) was used for the analysis of the light reaction products (H_2_, CH_4_). Light hydrocarbons (C_2_–C_3_), water, and oxygenates were analyzed by another TCD coupled with a RT-Q-Bond precolumn (length 3 m; i.d. 0.32 mm; film thickness 10 μm) and an RT-Q-Bond column (Restek, length 10 m; i.d. 0.32 mm; film thickness 10 μm). Heavier hydrocarbons (C_4_ to trimethylbenzenes) were separated using an Rtx-1 column (Restek, length 15 m; i.d. 0.32 mm; film thickness 1 μm) and analyzed with a flame ionization detector (FID). Conversion was defined as the carbon-based fraction of oxygenates (methanol and dimethyl ether) consumed during the reaction. Selectivity to products was calculated on the carbon atom basis.^21^ Further experimental details are provided in SI.

### Step-Response Reaction Experiments

We adapted the scanning pulse-gas chromatography method describe previously^15^ for periodic step-response experiments. The approach is schematically illustrated in Figure S5. In a typical experiment, we regularly switched between dry He and MeOH-containing He flows using a 4-way valve controlled by a home-built timer. A thermostated saturator was used to supply methanol vapor to the catalyst bed: a He flow of 5 mL·min^–1^ was led through a saturator containing liquid methanol, which was kept at −14.6 °C. Ar was introduced as an internal standard and tracer to the MeOH-containing He flow at a rate of 5 mL·min^–1^, resulting in a methanol partial pressure of 0.75 kPa. The dry He flow was fed at a rate 10 mL·min^–1^ to the catalyst bed. Calibrated thermal mass-flow controllers (Brooks) were used to supply the gases to the reactor. The reactor outlet was connected to a GC (Compact GC 4.0, Global Analyzer Solutions) and MS (Pfeiffer Omnistar) via heated gas transfer lines. The total GC analysis time was 432 s. The scheme of the setup is provided in Figure S6.

A fixed catalyst amount of 50 mg was used during the switching feed experiments. In a typical experiment, pelletized (250–500 μm) catalyst was pretreated at 550 °C in a flow of artificial air for 1 h and then cooled to 350 °C in He flow. Then, the first switch to methanol was performed, and the online analysis by GC and MS was started. After, the feed was switched from methanol-containing He flow to dry He flow every 1098 s (±10 s) and samples of the reactor effluent were injected into the GC every 432 s (±1 s). As a result of the asynchronous switching and GC injections, an array of about 100 chromatograms, corresponding to different points along the 36.6 min MeOH in He/dry He cycle, was obtained. Data processing involved assigning each chromatogram to a specific time after the switch was performed, which is further referred to as “time on switch”. We excluded the first 1–2 switches, where unstable response to the methanol feed was observed, and verified that all the methanol switches thereafter led to identical product distribution with MS analysis. The detailed procedure for quantification of the step-response results is provided in SI.

### Photoelectron Photoion Coincidence Spectroscopy

Photoionization experiments in the range 9.0–11.5 eV were carried out at the DESIRS undulator beamline of the SOLEIL synchrotron facility (Paris, France) using the SAPHIRS molecular beam end station^22^ equipped with the double-imaging photoelectron photoion coincidence spectrometer (i2PEPICO) DELICIOUS III.^23,24^ The beamline was set to deliver a photon flux of the order of 10^12^ photons·s^-1^, and two different monochromator slits were used, with resolutions 100 (16 meV dE) and 600 (38 meV dE) μm. Spectral purity was ensured by a windowless gas cell filled with argon to remove contributions from the high harmonics of the undulator.

A homemade flow tube reactor was connected to the 3D manipulator axis of the SAPHIRS expansion chamber (Figure S7). In a typical experiment, 100 mg of the catalyst powder was pretreated at 550 °C in a flow of artificial air for 1 h and cooled to 350 °C in He flow. Then, the methanol vapor was supplied to the reactor (12.3 kPa of MeOH in 75 mL·min^–1^ He). A sampling nozzle was connected to the outlet of the reactor tube, and the gas containing reactants and products was adiabatically expanded and skimmed twice. The resulting molecular beam crossed the ionizing light beam at the center of DELICIOUS III, and the produced ions and electrons are accelerated in opposite direction by a constant electric field of 90 V·cm^-1^ and analyzed by a Wiley–McLaren time-on-flight (TOF) mass spectrometer and a velocity map imaging (VMI) device, respectively. When ionization is performed with continuous sources such as synchrotron radiation, the ionization events are well separated in time, and the electron and ion from the same ionization event can be correlated, in what is called coincidence detection. The mass-selected cation spectroscopy is obtained by scanning the photon energy and recording the coincidence signal as a function of photon and electron energy to yield the mass-selected threshold photoelectron spectra (ms-TPES), as described elsewhere.^25,26^ In this work, ms-TPES were recorded with total energy resolutions of 100 (16 meV dE) and 600 (38 meV dE) μm in the energy range 9–11.5 eV for the detection of all observable intermediates at 350 °C reaction temperature.

### Operando TGA-MS

To quantitatively assess the amount of reactive species formed and retained over working catalysts, we performed operando TGA-MS measurements. For this purpose, we used Mettler Toledo TGA/DSC 1 instrument connected to a mass spectrometer (Pfeiffer Omnistar). A catalyst amount of 10 mg was placed in an alumina crucible and pretreated in O_2_:He (1:3 vol. ratio) flow at 550 °C (heating rate 10 °C·min^–1^) for 1 h to remove contaminants, followed by cooling down to reaction temperature 350 or 450 °C. After that, TG analysis was started. In a typical experiment, we used an automated 4-way valve to switch the feed every 20 min from dry He to MeOH-containing flow (Figure S8). A thermostated saturator was used to supply methanol vapor to the catalyst bed: a He flow of 40 mL·min^–1^ was fed through the saturator followed by dilution with another He flow of 40 mL·min^–1^. The temperature of the thermostat was kept at −14.6 °C, resulting in a methanol partial pressure of 0.75 kPa (after the dilution with a side He flow of 40 mL·min^–1^). A pure He flow of 80 mL·min^–1^ was supplied to the catalyst bed during the dry phase. Calibrated thermal mass-flow controllers (Brooks) were used to supply the gases to the TGA chamber.

### MAS NMR

Solid-state magic angle spinning nuclear magnetic resonance (MAS NMR) spectra were recorded using an 11.7 T Bruker NMR spectrometer operating at 500 MHz, 125 MHz, and 130 MHz for ^1^H, ^13^C, and ^27^Al, respectively. ^1^H and ^13^C MAS NMR experiments were performed using a Bruker triple channel 4 mm MAS probe head spinning at rates between 8 and 10 kHz. Prior to ^1^H measurements, the samples were dehydrated and sealed in air- and moisture-free glovebox. One-dimensional ^13^C{^1^H} cross-polarization (CP) and two-dimensional ^1^H–^13^C{1H} HETCOR (HETeronuclear CORrelation) MAS NMR spectra were recorded with a ramped contact pulse time of 5 ms and an interscan delay of 3 s. ^13^C direct excitation (DE) spectra were measured using a high power proton decoupling Hahn echo pulse sequence p1-τ1-p2-τ2-aq with a 90° pulse p1 = 5 μs, a 180° pulse p2 = 10 μs, and an interscan delay of 10 s. ^13^C NMR spectra were recorded at a spinning rate of 8–10 kHz. ^27^Al NMR spectra were recorded using a Bruker 2.5-mm MAS probe head spinning at 25 kHz.NMR shift calibration for ^1^H, ^27^Al, and ^13^C was done using tetramethylsilane (TMS), saturated Al(NO_3_)_3_ solution, and solid adamantane, respectively.

### Operando IR Spectroscopy

#### Methanol Step-Response Experiments

To study the response of the catalysts to the switches between methanol and dry He by IR spectroscopy, we used a Bruker Vertex 70v IR spectrometer and adapted the setup for methanol dosing (Figure S9). Spectra were taken in the 4000–1000 cm^–1^ range. Samples were pressed into self-supporting wafers (10–20 mg, diameter 1.3 cm) and placed in an environmental cell. The wafers were pretreated in O_2_:He (1:2 vol. ratio) flow at 550 °C (heating rate 10 °C·min^–1^) to remove contaminants followed by cooling to 350 °C in He. After that, IR analysis was started. First, background spectra were recorded. In a typical experiment, we used an automated 4-way valve to switch the feed every 20 min from dry He to a MeOH-containing He flow. A thermostated saturator was used to supply methanol vapor to the catalyst bed: a He flow of 10 mL·min^–1^ was led through the saturator followed by dilution with another He flow of 120 mL·min^–1^. The temperature of the thermostat was kept at −14.6 °C that after dilution with a side He flow of 120 ml·min^-1^ resulted in a methanol partial pressure of 0.12 kPa. A dry He flow at a rate of 130 mL·min^–1^ was used when no methanol was fed to the sample. Calibrated thermal mass-flow controllers (Brooks) were used to supply the gases to the IR cell. The outlet of the IR cell was connected to an MS (Pfeiffer Omnistar MS).

#### Temperature-Programmed Experiments with Water

To perform temperature-programmed experiments with water, the same methodology was used. In a typical experiment, we increased the temperature from 150 to 500 °C with heating rate 5 °C·min^–1^ while feeding water and recording IR spectra simultaneously. A thermostated saturator was used to supply water vapor to the catalyst bed by evaporation in a He flow at a rate of 10 mL·min^–1^ and diluted by a side He flow of 130 mL·min^–1^. The temperature of the thermostat was kept at 7 °C, resulting in a water partial pressure of 0.07 kPa (after the dilution with side He flow of 130 mL·min^–1^).

#### Operando XRD

Operando XRD experiments were performed at the ID31 beamline of ESRF synchrotron (Grenoble, France). The photon wavelength was 0.0165 nm (75 keV) with an unfocused beam of 0.5 mm × 0.5 mm, and a Pilatus3 X CdTe 2 M X-ray detector (Dectris) was used. Sieved (250–500 μm) ZSM-5 catalyst (20 mg) was placed in a quartz capillary (i.d. 2.8 mm, o.d. 3.0 mm, wall thickness 0.1 mm) to form a catalyst bed of 4 mm in length. The capillary was sealed by PTFE ferrules in a home-built Clausen-type flow cell,^27^ located on a movable sample stage. The catalyst bed was heated to 550 °C using two gas blowers (Cyberstar) for the pretreatment in artificial air flow 50 mL·min^–1^ for 1 h and then cooled down to 400 °C (reaction temperature). The temperature was controlled by a thin (0.25 mm) K-type thermocouple placed inside the catalyst bed. After the temperature reached 400 °C and was stabilized for 30 min, blank diffractograms were recorded, and 50 mL·min^–1^ He flow along with evaporated methanol (13 kPa) and Ar tracer 2 mL·min^–1^ was supplied to the catalyst bed. The chemical composition of the outlet flow was analyzed by a quadrupole mass-spectrometer (QGA, Hidden Analytica). The capillary was moved up and down during the experiment to acquire diffractograms at different positions of the bed. The integrated XRD patterns were analyzed by Rietveld refinement using the GSAS-II software. The patterns were refined in *q*-range of 0.4–6 Å^–1^. The scale factor, background, and the unit cell parameters (*Pnma* space group) were refined. Other parameters (crystal size, strain, displacement, atomic and thermal parameters, etc.) were initially refined for the HZSM-5 sample before switching to methanol and kept the same for each pattern during the following refinement.

## Results and Discussion

### Catalytic Performance

**Figure 1:**
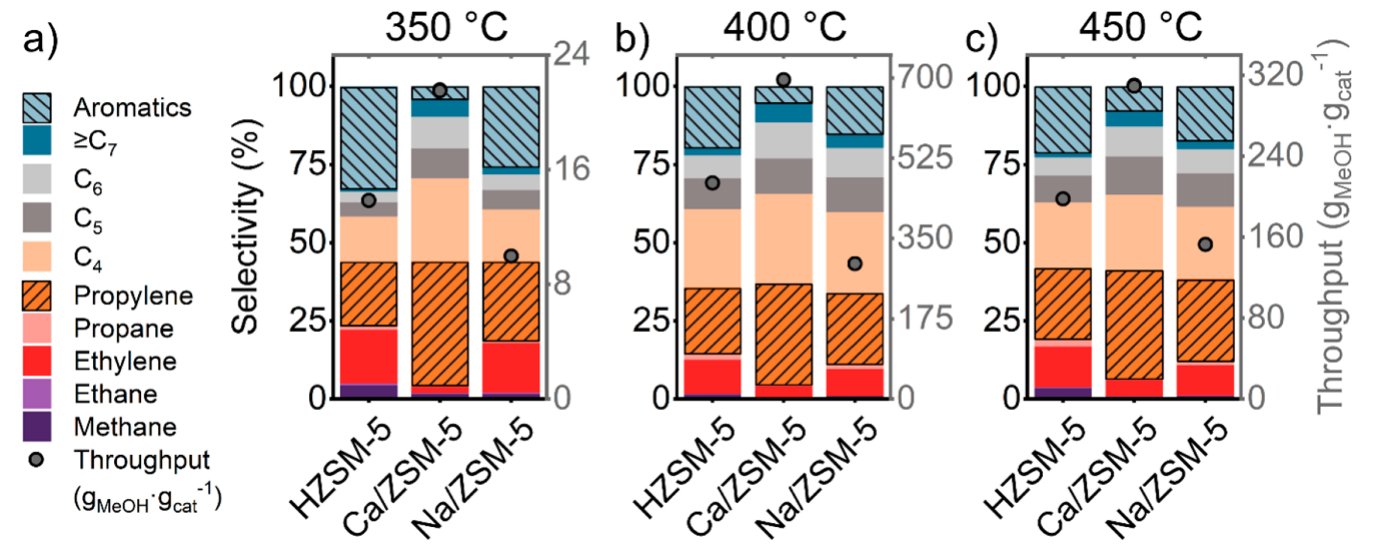
Integral carbon selectivity and methanol throughput values calculated until the moment when conversion of methanol drops below 75%. Reaction conditions: (a) 350 °C, 25 mg of catalyst, 0.75 kPa of MeOH, carrier −10 mL·min^–1^ He, WHSV 0.3 h^–1^; (b) 400 °C, 25 mg of catalyst, 6 kPa of MeOH, carrier −30 mL·min^–1^ He, WHSV 6 h^–1^; and (c) 450 °C, 25 mg of catalyst, 12.3 kPa of MeOH, carrier −30 mL·min^–1^ He, WHSV 12 h^–1^.

First, we compared the catalytic performance of Ca/ZSM-5 against the two reference catalysts HZSM-5 and Na/ZSM-5 in a range of reaction temperatures and space velocities. The BAS density distribution in zeolite affects the selectivity of the methanol-to-hydrocarbons reaction – a higher density of acid sites favors the production of aromatics, while a lower acid site density usually leads to a higher selectivity of C_3+_ olefins.^28^ Here, we compared the effect of decreasing the acid site density in the parent HZSM-5 zeolite using Ca^2+^ and Na^+^ cations. This comparison allows us to keep all other properties of the samples (crystal size, number of silanol defects, etc.) similar. In this way, we could decouple the effect of decreased acid site density and Ca-modification. The presence of Ca^2+^ leads to enhanced formation of C_3_ – C_7_ hydrocarbons (mostly olefins) at all three reaction temperatures applied here (Figure 1). The selectivity to propylene is particularly enhanced by Ca-modification. The total methanol throughput was substantially higher for Ca/ZSM-5 catalyst as well. The higher propylene selectivity, improved catalyst lifetime, and decreased aromatics selectivity upon modification with Ca^2+^ are in line with previous reports.^9−11,13,29^

**Figure 2:**
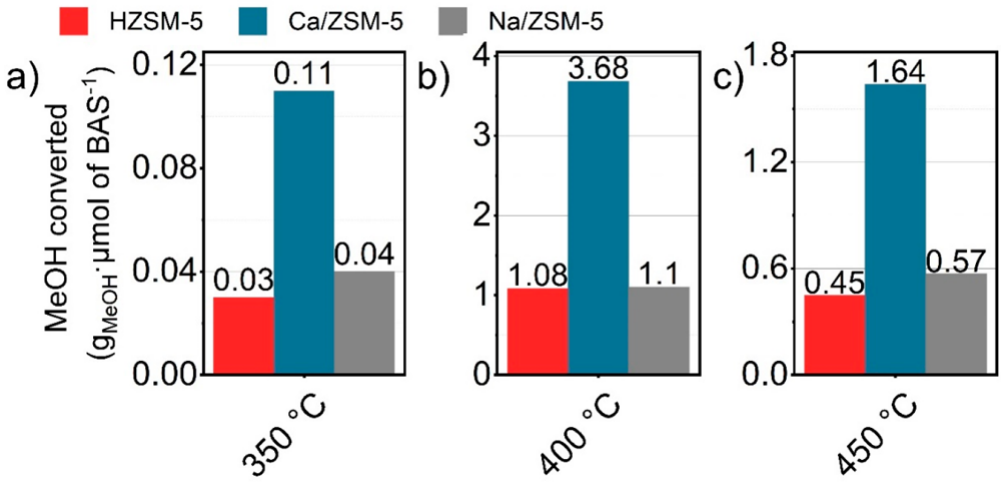
Total amounts of methanol converted normalized by number of BAS for respective catalysts.

It is common practice to relate the methanol throughput to the number of Brønsted acid sites.^30,31^ For the current experiments we note that, whereas the total numbers of methanol molecules converted per acid site are similar for HZSM-5 and Na/ZSM-5, this value is much higher for Ca/ZSM-5 (Figure 2).

As in addition to acid sites, hydrocarbon pool intermediates play an important role as active sites in the MTH mechanism,^14^ these results point to a likely different distribution of hydrocarbon pool components between HZSM-5 and Na/ZSM-5 on the one and Ca/ZSM-5 on the other hand. In order to understand the differences in reaction selectivity and stability, it is necessary to know the detailed composition of reaction products, intermediates, and hydrocarbon pool components.

### Analysis of Reaction Products and Intermediates by PEPICO

**Figure 3:**
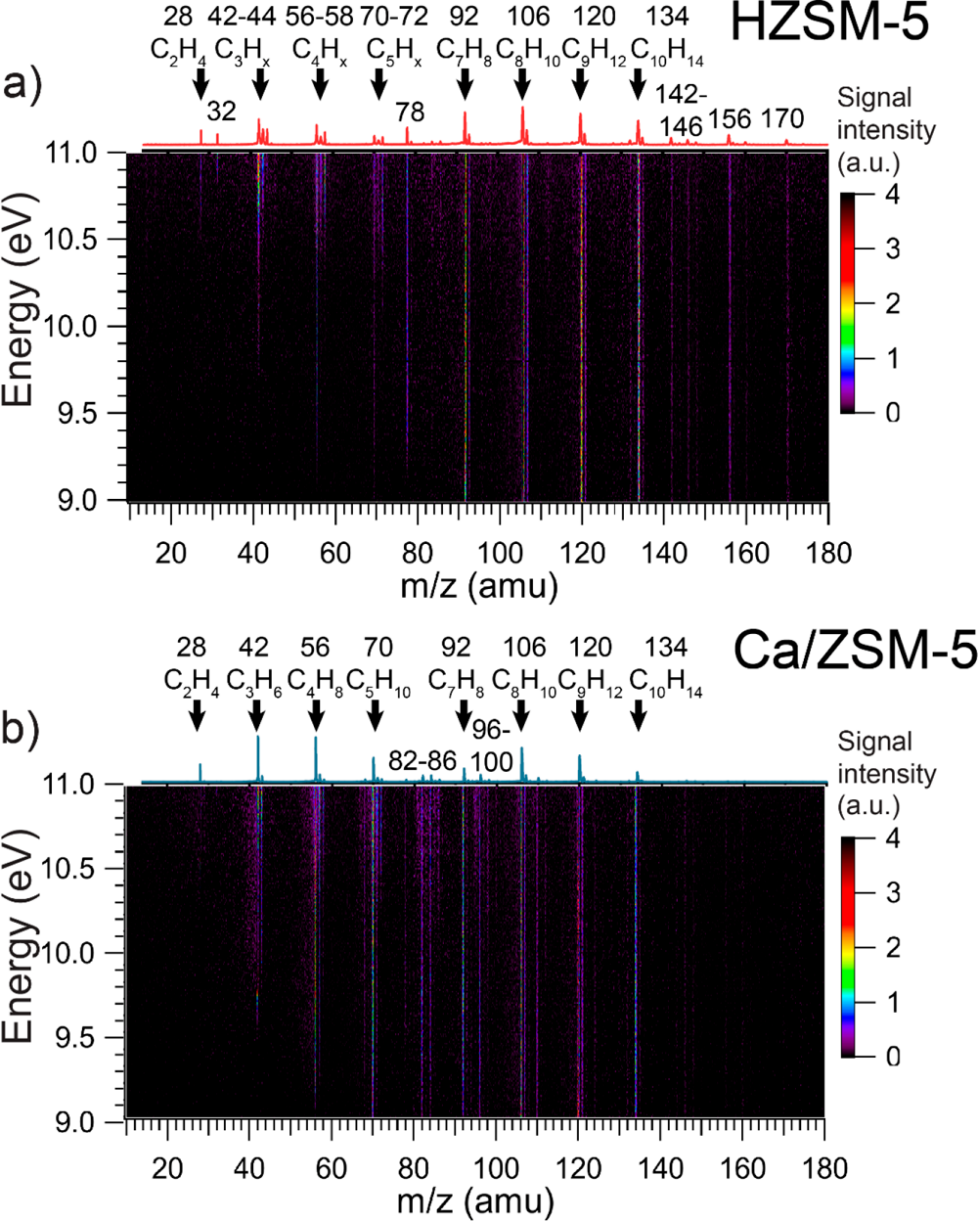
Ion signal as a function of *m*/*z* and photon energy from the detected products and intermediates over HZSM-5 (a) and Ca/ZSM-5 (b) catalysts during the MTH reaction. Reaction conditions: 350 °C; 100 mg of the catalyst, 12.3 kPa of methanol, carrier −75 mL·min^–1^ He. The data acquisition took 8 h for each catalyst.

From the lower amount of C_1_–C_3_ paraffins (Figure 1), we infer that Ca^2+^ ions suppress hydrogen transfer reactions. Complementary to conventional GC analysis, we used PEPICO to study other possibly short-lived reaction products obtained over HZSM-5 and Ca/ZSM-5. This technique has been successfully used before in the mechanistic analysis of various catalytic reactions.^32−34^Figure 3 shows the evolution of the mass spectra as a function of photoionization energy. We first performed a wide scan of the reactor eluent with incident energies in a range of 9.0–11.5 eV and recorded threshold photoionization matrix for the detection of all observable intermediates and products formed during methanol conversion (Figure 3). The results show that the catalysts produce the typical C_1_–C_10_ aliphatic and aromatic hydrocarbons also observed by GC analysis. Stronger signals related to aromatics were observed for HZSM-5, in particular heavy products (*m*/*z* = 142–146, 156, 170), which were not detected with our GC. These molecules can be assigned to methylated naphthalenes with different hydrogenation degrees^35^ and with different number of methyl groups (one −CH_3_ group for 142–146 signals, two for 156, and three for 170).^35,36^ In contrast, together with the absence of signals of heavy polyaromatic compounds, more intense signals corresponding to various aliphatic compounds were observed for Ca/ZSM-5.

**Figure 4:**
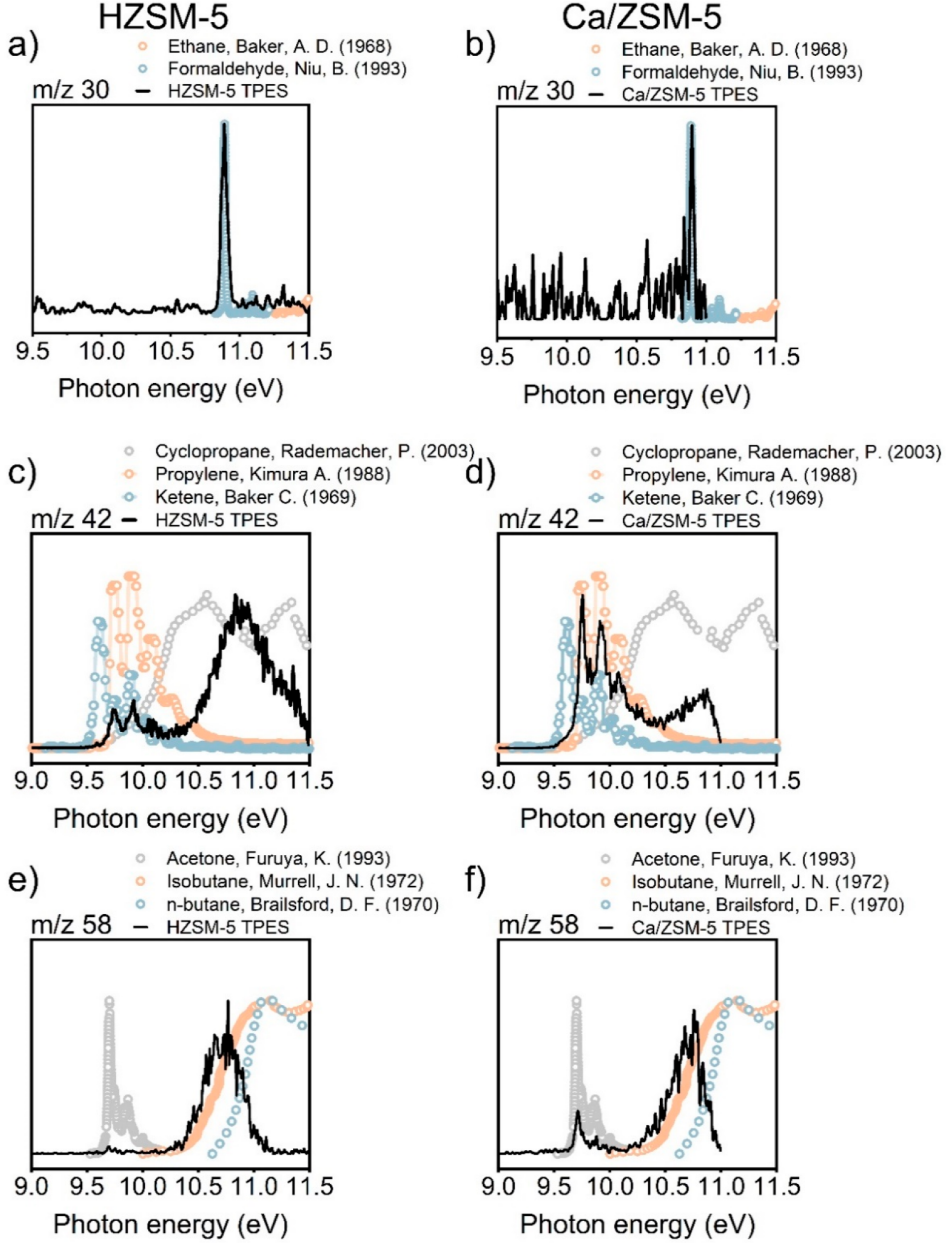
Comparison of recorded and literature photoelectron spectra^42−48^ for selected *m*/*z* = 30, 42, and 58 channels over HZSM-5 (left) and Ca/ZSM-5 (right), corresponding to various oxygenate and hydrocarbon species. Reaction conditions: 350 °C, 100 mg of the catalyst, 12.3 kPa of methanol, carrier −75 mL mL·min^–1^ He.

For each of the mass peaks observed in Figure 3, ms-TPES can be extracted, some of which are plotted in Figure 4. The results indicate the formation of several unstable oxygenate intermediates.^32^ For example, we detected formaldehyde (*m*/*z* = 30, Figure 4a,b), one of deactivating species formed from methanol disproportionation, which is difficult to detect with GC analysis.^31,37,38^ A much stronger signal of formaldehyde was observed for HZSM-5 in comparison to Ca/ZSM-5. The lower production of formaldehyde, likely caused either by its decomposition over Ca cations or the lower Brønsted acidity of Ca/ZSM-5, can explain the slower deactivation observed for Ca/ZSM-5.^39,40^ Similar results, showing suppressed formaldehyde production over Ca-modified zeolites, were reported by Paunović et al.^41^ For ms-TPES with *m*/*z* 42, lack of signal at 9.6 eV shows that the yield of ketene is negligible with respect to that of propylene for both HZSM-5 and Ca/ZSM-5. At the same time, we observed more acetone (*m*/*z* = 58), over Ca/ZSM-5. This finding may indicate that carbonylation reactions, which are generally considered to lead to the formation of the first C–C bonds in MTH chemistry, are not strongly affected by Ca^2+^ ions.^33^ As C_2+_ oxygenates were suggested to be active intermediates in the formation of C_5_ hydrocarbons, the formation of significant amount of C_5_ products over Ca/ZSM-5 (Figure 1) is not surprising.^33^ To summarize, combining catalytic testing, photoionization mass spectrometry, and ms-TPES, we demonstrated that the MTH product distribution is shifted toward aliphatics in the presence of Ca^2+^. The strong decrease in aromatic selectivity, with the production of heavier polyaromatics being particularly affected, might be linked to the suppressed formaldehyde production after modification with Ca.

### What Is Inside the Zeolite Pores during the Reaction?

#### Transient Kinetic Experiments

Kinetic experiments in continuous flow of methanol shed light on steady-state product distribution, but they cannot provide detailed information about the hydrocarbon pool composition and do not allow distinguishing molecules actively participating in the reaction from deactivating and spectator species.^49,50^ In an attempt to distinguish intermediates responsible for the shift in the reaction selectivity from the spectator and deactivating species, we employed step-response experiments, where a methanol-containing reaction feed was quickly replaced by dry He. The response of catalysts to these switches was monitored by GC analysis, thermogravimetry, infrared spectroscopy, and X-ray diffraction.

**Figure 5:**
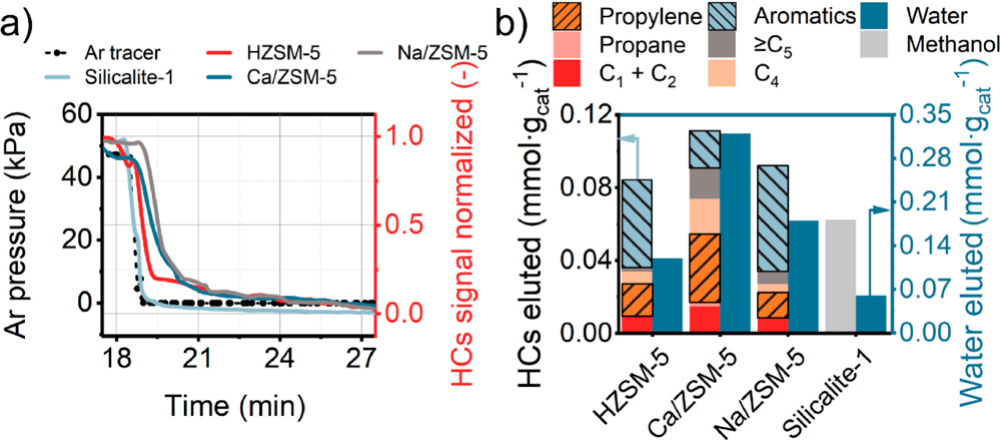
Step-response MTH experiments performed at 350 °C. (a) Total hydrocarbon traces of all catalysts compared to Ar tracer. (b) Amount of hydrocarbons eluted from zeolites after methanol was switched off. Conditions: 350 °C, 50 mg of catalyst, 18.3 min switch, 5 mL·min^–1^ He flow with 1.5 kPa of MeOH + 5 mL·min^–1^ side flow of Ar tracer.

To study the effect of Ca^2+^ on the composition of molecules, confined inside the zeolite pores, we used the scanning pulse gas chromatography (SP-GC) method described previously.^15^ We modified this method, so that the strength of the interaction of water and hydrocarbons with the catalyst can be gauged, providing quantitative information on the amount of mobile hydrocarbons and water retained in the zeolite pores during the reaction. The experiments consisted of identical periodic switches between a flow containing methanol and Ar tracer balanced by He to a dry He flow (Figures 5, S5–S6, and S10) followed by GC analysis. The switches and GC injections were asynchronized in such a way that each GC injection probed different time points after each switch. After ca. 30 identical switches, we could reconstruct one GC-derived switch profile with a time resolution of ca. 20 s and high chemical resolution. We integrated the difference between the Ar tracer response and signals of reaction eluents (water and hydrocarbon products) to estimate the amount of these molecules retained over the working catalyst. The experiments demonstrate the stronger retention of mobile hydrocarbon and water molecules over Ca/ZSM-5 during the reaction in comparison to HZSM-5 and Na/ZSM-5 (Figures 5a and S10). Contour maps reconstructed from the FID signal clearly illustrate the differences in product distribution. In line with the continuous flow catalytic results and PEPICO data, which showed suppressed aromatics formation over Ca/ZSM-5, the intensity of the GC peaks related to aromatics is lower for Ca/ZSM-5 after the methanol-containing feed was switched to dry He (Figure S12). In contrast, intense signals of aromatic eluents are observed for HZSM-5 and Na/ZSM-5. By inspecting the individual products, more propylene (as a product of olefin cycle) and much less toluene (as a product of aromatic cycle) were observed for Ca/ZSM-5 catalyst after methanol was switched off (Figure S11). The total yield of eluents after the switch to dry He was the highest for Ca/ZSM-5 catalyst followed by Na/ZSM-5 (Figure 5b). We should note that our GC does not allow analyzing hydrocarbons heavier than C_9_, such as tetramethylbenzenes and naphthalenes. Therefore, we cannot estimate their contribution in these transient experiments. Overall, the step-response data demonstrate that (i) Ca/ZSM-5 retains a much larger amount of water and a significantly larger amount of hydrocarbons than HZSM-5 and Na/ZSM-5 during the MTH reaction; (ii) the hydrocarbon pool intermediates inside the pores of Ca/ZSM-5 that can leave the pores are mostly aliphatic in nature; and (iii) HZSM-5 and Na/ZSM-5 contain more aromatic hydrocarbon pool species.

#### TGA-MS

**Figure 6:**
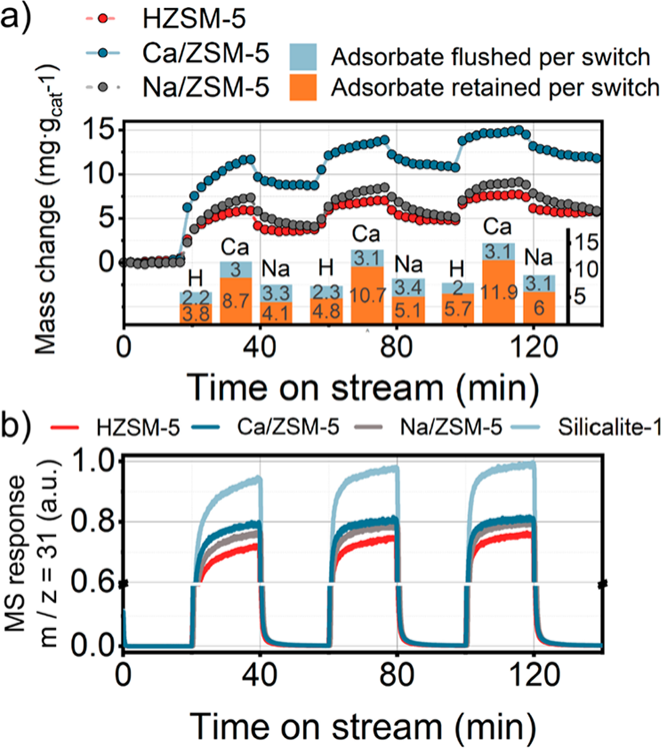
Operando TGA profiled recorded during switching from methanol-containing He flow to dry He over HZSM-5, Ca/ZSM-5 and Na/ZSM-5 catalysts. (a) TGA profiles and amount of adsorbates retained and flushed during three methanol switches for ZSM-5 catalysts (inset). (b) MS profiles of *m*/*z* = 31 signal corresponding to methanol recorded simultaneously with TG profiles. Conditions: 350 °C, 10 mg of catalyst, carrier −80 mL·min^–1^ He, 0.75 kPa of MeOH.

Complementary to the above transient experiments that allow analysis of intermediates and products that can leave the catalyst pores, we used TGA-MS to investigate species that are strongly retained by the catalysts. The corresponding data following a switch from the methanol-containing feed to dry He are given in Figures 6 and S15a. The results show that the Ca/ZSM-5 catalyst retains a much higher amount of adsorbates than HZSM-5 at similar conversion levels (Figures 6a, inset, and S15b). The total amount of retained molecules after just two switches was 1.4 wt % based on the initial catalyst weight. This amount corresponds to at least 10% of the micropore volume being occupied after the methanol was switched off (assuming average density of adsorbed molecules is 1 g·cm^–3^). It is important to note that Na/ZSM-5 also retained a larger amount of adsorbates during methanol conversion than HZSM-5, but these molecules desorbed after the methanol flow was switched to He. The desorption rate from Na/ZSM-5 was slow, so we hypothesize that these slowly desorbing adsorbates are larger polyaromatic species that were not observed in the transient experiments using GC analysis. Altogether, the transient GC and TGA-MS results suggest that a much higher amount of immobile species are retained in Ca/ZSM-5 as compared to Na/ZSM-5 and HZSM-5. These immobile species occupy a significant fraction of the microporous space, which can influence the formation of hydrocarbon pool components.^20^ By performing catalytic and TGA-MS experiments for samples with varying Ca^2+^ loading (Figure S16 and Table S2), we found that there is an optimum in the Ca^2+^ loading. Above a Ca content of 1 wt %, the concentration of retained adsorbates becomes too high to allow the MTH reaction to proceed, likely because the formation of hydrocarbon pool species is severely hindered (Figure S16b).

#### MAS NMR Study of Hydrocarbons Occluded in the Pores

**Figure 7:**
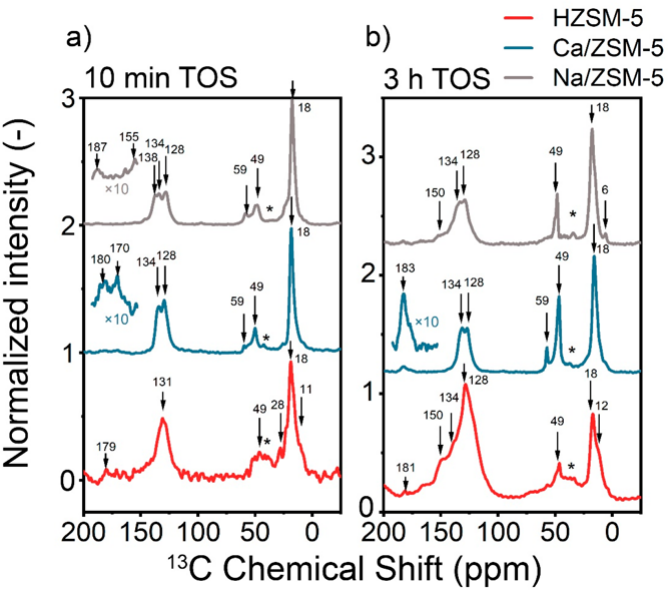
^1^H–^13^C CP MAS NMR of the used catalysts from continuous flow experiments after 10 min (a) and 3 h (b) on stream. Conditions: 350 °C, 100 mg of the catalyst; carrier −30 mL·min^–1^ He, 12 kPa of ^13^C MeOH. Sidebands are denoted with asterisks.

**Figure 8:**
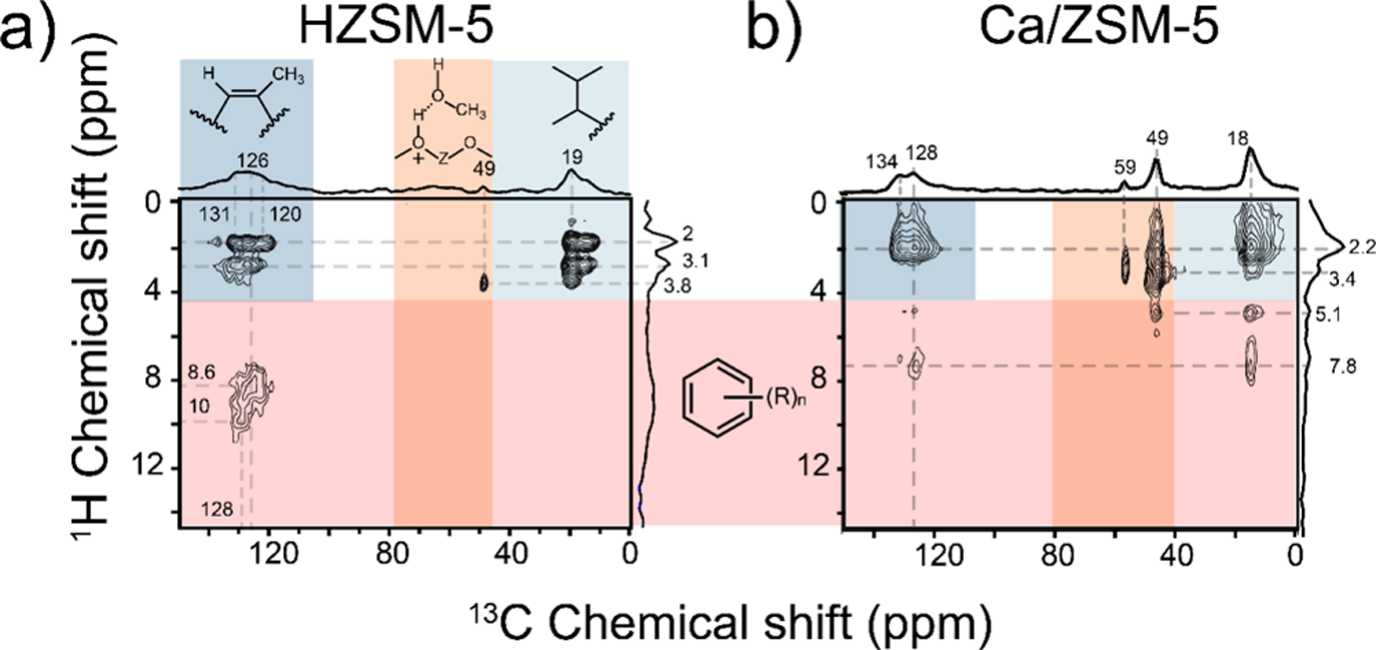
^1^H–^13^C{^1^H} HETCOR MAS NMR 2D of the used catalysts from continuous flow experiments after 3 h of stream. Conditions: 350 °C, 100 mg of catalyst, carrier −30 mL^–1^·min He, 12 kPa of ^13^C MeOH.

The change in the product distribution upon introduction of Ca^2+^ is due to the different nature of the active hydrocarbon pool species.^51^ To study this aspect, we carried out ex situ MAS NMR experiments of used catalysts to understand the structure of molecules occluded inside the catalyst pores (Figures 7–8 and S17–S18). These experiments revealed a significant difference in the type and amount of hydrocarbon species retained after 10 min on methanol stream (Figure S17). The ^13^C NMR signals between 0 and 30 ppm can be assigned to aliphatics, those in the range of 120–150 ppm to aromatics and olefinic species, and the signal at 49 ppm is due to adsorbed methanol.^52,53^ Ca/ZSM-5 presents stronger relative signals of oxygenate species than the other two catalysts. Even after 3 h on stream, the amount of aromatics in the pores of Ca/ZSM-5 is much lower in comparison to HZSM-5 (Figure S18). In addition to a strong signal of methanol, the spectrum of used Ca/ZSM-5 after 3 h reaction features a signal at 59 ppm, which can be assigned to adsorbed dimethyl ether and methoxy species.^52−54^ The assignment of the signals is provided in Table 2. The higher amount of oxygenates is in line with the TGA results, which showed that lighter adsorbates prevail for the Ca/ZSM-5 sample after 3 h on stream, while heavier coke species with a higher combustion temperature are formed over HZSM-5 (Table S3 and Figure S19).^55^

**Table 2:** Assigned Signals from ^1^H–^13^C NMR Profiles, Corresponding to the Adsorbates Deposited on the Catalyst

Correlation peaks (ppm)	Assignment	Source
^13^C nucleus		
6, 10, 12, 18	–CH_2_	(56, 57, 58, 59, 60, 61)
	–CH_3_ (methyl groups on the benzene rings)	
28, 29, 43	–CH or methyl groups	(56, 62)
49	Adsorbed methanol molecules	(52, 53)
59	Adsorbed DME molecules or methoxy species	(52, 53)
118–135	Olefinic species	(12, 58, 59, 63, 64, 65, 66)
131, 128, 134	Polymethylbenzene species, −C of aromatic hydrocarbons	(56, 57, 58, 59, 61, 62, 66, 67, 68, 69, 70)
138, 140	Polymethylbenzene species, −C of polyaromatic hydrocarbons	(56, 68, 69, 71, 72)
	Methylbenzenes, alkyl-substituted aromatics	
150, 154	Polyaromatics	(73,74)
179, 181, 183, 184	Carbonyl groups	(75)
^1^H nucleus		
0.4–1.1	H_α_ on aromatic ring/terminal CH_3_	(74)
1.1–2.05	H_β_ to aromatic/in paraffinic CH and CH_2_	(74)
2.05–4.5	H_γ_ to aromatic ring	(74)
6–10	Aromatic protons	(59, 66, 74)

#### Operando IR

**Figure 9:**
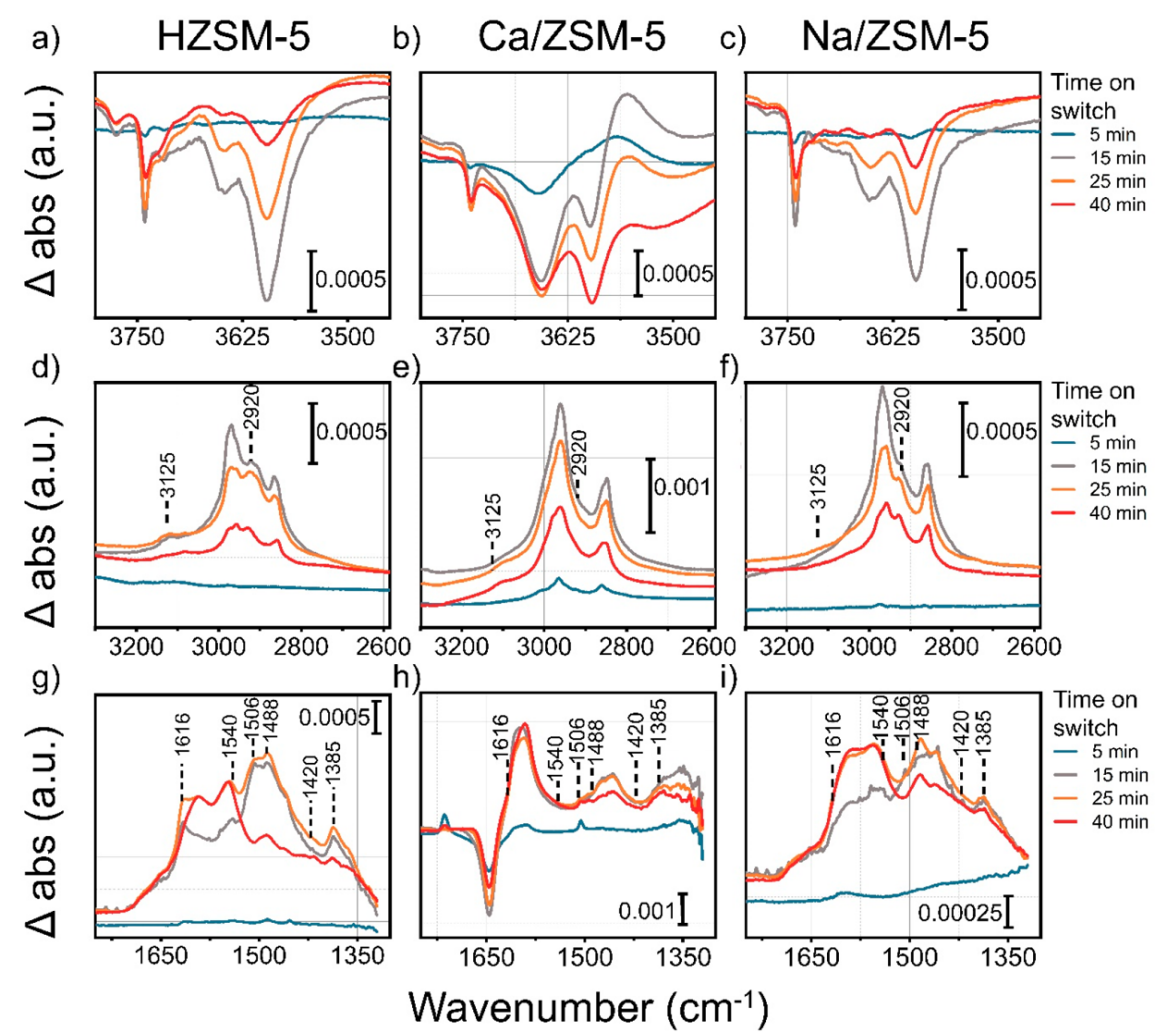
Enlarged regions of Δ abs spectra: (a–c) OH-region and (d–f) region of methanol adsorption, and (g–i) 1750–1300 cm^–1^ region. Δ abs spectra were obtained by subtraction of the first spectrum recorded at 350 °C in absence of methanol from all other spectra. Conditions: 350 °C, 15 mg of catalyst pellet, carrier −130 mL·min^–1^ He, 0.12 kPa of MeOH.

**Figure 10:**
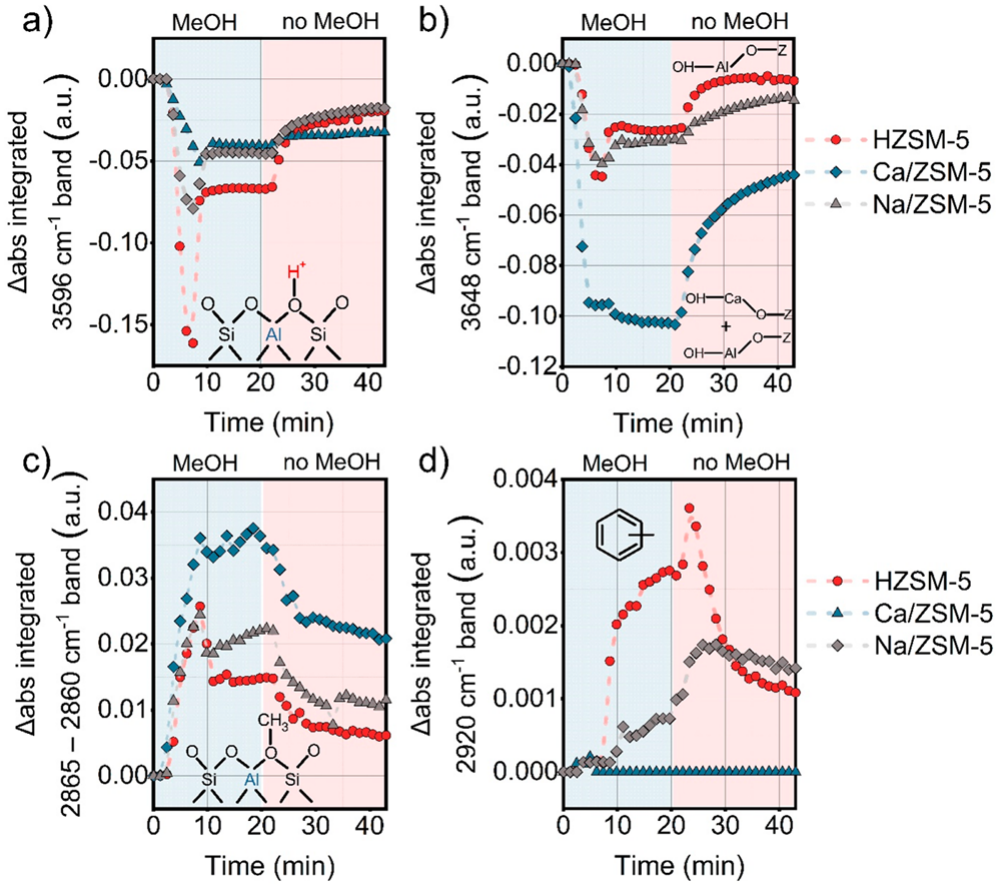
Bands of interest integrated from Δ abs spectra from operando IR measurements for ZSM-5 catalysts in presence and absence of the methanol feed: (a) BAS, (b) EfAl and Ca-OH, (c) methoxy groups, and (d) polymethylated benzenes. Conditions: 350 °C, 15 mg of catalyst pellet, carrier −130 mL·min^–1^ He, 0.12 kPa of MeOH.

Having found that the Ca^2+^ species strongly affect the amount and the nature of retained adsorbates, we turned to transient operando IR experiments to explore the interaction of these adsorbates with zeolite during the reaction. These studies included step-response methanol switches and temperature-programmed experiments with water vapor. First, we recorded IR spectra upon switches between methanol-containing He and dry He flows, while analyzing the cell effluent by MS (Figures 9 and S20). We observed that initially bands belonging to OH groups were rapidly replaced by bands related to adsorbed methanol. For the HZSM-5 catalyst with the highest density of OH groups, methanol molecules tend to chemisorb on BAS (3596 cm^–1^), forming methoxy groups which can participate in the initiation of hydrocarbon pool (Figure 10a,c).^76^ 2D correlation maps^77^ from IR spectra, recorded during switching between methanol-containing and dry He flows (Figure S21), emphasize the strong correlation between bands of adsorbed methanol and the bridging hydroxyl groups for HZSM-5 and Na/ZSM-5. We observe that OH bands sequentially disappear while new bands with positive intensity appear (cf. 3596 cm^–1^, 3650 cm^–1^, 3710 cm^–1^, 3741 cm^–1^ vs 2750–3000 region, respectively, Figure S21a–c). Comparing the evolution of the OH bands in the 3630–3650 cm^–1^ range, which represent OH groups connected to extraframework Al species (Al–OH, EFAl)^78^ and, additionally, Ca^2+^ for Ca/ZSM-5^79^ (Figure 10b, Figure S23a), the largest intensity decrease was observed for Ca/ZSM-5. This suggests enhanced interaction of methanol with Ca^2+^ via OH-bridging. Previous studies assigned the 3640 cm^–1^ band to Ca–OH in Ca-modified zeolites.^80,81^

After about 10 min in methanol flow, a functional hydrocarbon pool is developed over all catalysts, as manifested by the stable formation of hydrocarbon products (signals *m*/*z* = 41, 57, 91 for propylene, butane and toluene in Figure S20b,d,f, respectively) and partial recovery of BAS (Figure 10a).^76^ Methoxy groups at 2865–2860 cm^–1^, which participate in the initiation of the C–C formation,^16^ are partially consumed in all catalysts (Figure 10c) after 10 min on stream. Several bands such as those at 1386, 1474, 1496, 1506, 1540, 1580, 1593, 1616 cm^–1^ related to ν(C–H), ν(C=C), and δ(C–H) vibrations indicate the formation of olefinic and (methylated) aromatic species during the reaction. Bands at 2920 and 3125 cm^–1^ due to (methylated) benzenes and cyclopentenyl cations, observed for HZSM-5 and Na/ZSM-5, are absent for Ca/ZSM-5 (Figures 9 and 10d). These findings point to the similarity of the reaction pathways for HZSM-5 and Na/ZSM-5 and their distinct difference with Ca/ZSM-5.

When methanol was switched off, strongly adsorbed species were retained over the catalysts (20–40 min on stream). For Ca/ZSM-5, these species were mainly methoxy groups, adsorbed methanol, and water (1647, 2750–3000 cm^–1^). In contrast, for HZSM-5 and Na/ZSM-5, aromatics were some of the major retained species (1506 and 2920 cm^–1^, Figures 9 and 10d).^82,76^ This difference is in line with the ^13^C NMR data, which pointed to more oxygenates in used Ca/ZSM-5, and the predominance of methylated aromatics in used HZSM-5 and Na/ZSM-5 (Figures 7, S18, and Table S3). Methoxy groups and adsorbed methanol molecules were still present in Ca/ZSM-5 long after methanol was switched off, pointing to the strong interaction of Ca^2+^ species with these adsorbates (Figure 10c). The assignment of the bands observed in these experiments is based on literature (Table 3 and Figure S20a,c,e). We note that care should be taken in the assignment of the different hydrocarbons and oxygenates due to the strong overlap in C–H and C–C regions.^83^ Nevertheless, the spectra in the 1350–1500 cm^–1^ region are similar for HZSM-5 and Na/ZSM-5 samples and very different from that of Ca/ZSM-5 (Figure 9g,h,i). The main inference from these data is the much stronger interaction of Ca/ZSM-5 with methanol and water and suppressed formation of aromatics over this sample.

**Table 3:** Literature Assignment of Vibrational Bands Observed by Operando IR for ZSM-5 Catalysts upon Methanol Adsorption

Vibration	Notation	Wavenumber	Ref
OH groups bonded to EfAl		3770–3780	(84, 85)
Isolated silanol	ν[Si–OH]_iso_	3741	(78, 86)
Isolated silanol on defect Si	ν[Si_def_–OH]_iso_	3710	(78)
Isolated hydroxyl on EfAl	ν[Al_ef_–OH]_iso_	3650	(78)
Ca-OH groups		3630	(79−81)
Brønsted acid site (BAS)	ν[Si–O(H)–Al]	3596	(78)
BAS H-bonded		3550, 3385	(87)
Adsorbed water	ν[H–O–H], δ[H–O–H]	3200–3600, 1647, 1629	(88−90)
Allyl C–H of methylated cyclopentenyl cation		3125, 1485	(76,82)
Methoxy groups	ν[Si–O(CH_3_)–Al], δ[Si–O(CH_3_)–Al]	2980, 2968, 2865, 1457	(16, 78, 91)
Methoxy on extra framework Si	ν[Si_ex_–OCH_3_]	2957, 2854	(78)
Methanol preadsorbed	ν[CH_3_OH]	2955, 2845, 1710, 1420, 1346	(78, 91−93)
Polymethylated benzenes		2920	(82, 83)
Substituted single aromatics and its protonated counterpart		1616, 1488, 1420, 1385	(76, 82, 94− 96)
Ethylene preadsorbed	ν[C_2_H_4_]	1615, 1593	(97)
Methyl groups	δHCH_2_	1479, 1457, 1436, 1395	(82, 98, 99)
Coke or polyaromatics		1580, 1521	(100, 101)
Toluene		1496	(96)
Trimethylbenzenes		1506, 1474	(96)
Alkylnaphthalenes		1540	(102)
Aliphatics	δCH_2_	1386	(103)

**Figure 11:**
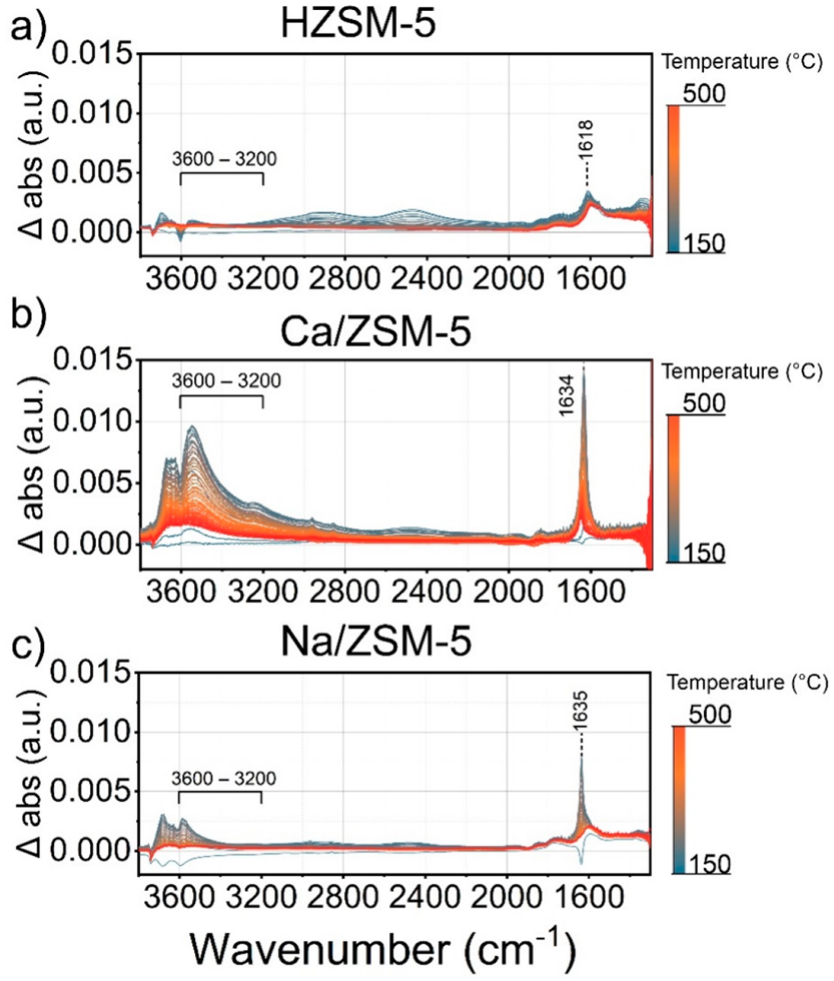
IR measurements of ZSM-5 catalysts combined with temperature-programmed experiments with water. Conditions: 150–500 °C, heating rate 5 °C·min^–1^, 15–20 mg of catalysts, carrier −130 mL·min^–1^ He, 0.07 kPa of water.

In addition to methanol switches, we studied the interaction of water with catalysts by IR spectroscopy upon exposure of catalysts to water vapor in a temperature-programmed regime. Ca/ZSM-5 retained a larger amount of water in a broad temperature range (Figures 11 and S22), indicating a much stronger interaction with water under reaction conditions. We also used IR spectroscopy of adsorbed pyridine^104^ on dry and wet samples (Figures S23–S24), where Ca/ZSM-5 catalyst showed a weaker intensity of pyridine bands upon adsorption of pyridine on wet samples. These results demonstrate that Ca/ZSM-5 has high affinity to water. We suggest that strongly adsorbed water molecules together with methanol, methoxy groups, and other adsorbates occupy the pores of Ca/ZSM-5, thereby changing the reaction environment. In summary, the results indicate that methoxy groups, methanol and water molecules are strongly retained by Ca ions. We speculate that this strong adsorption of water, methanol, DME, and methoxy groups on Ca^2+^ ions can interfere with the hydrocarbon pool chemistry, limiting the formation of bulky aromatics and favoring the olefin cycle.

#### Link between the Structural Properties of Catalysts and Retained Adsorbates

To extend the understanding of adsorbate effects on the structure of catalysts, we performed operando and ex situ XRD experiments supplemented by ex situ TGA.

#### Operando XRD

It is known that the polarity and size of adsorbate molecules can lead to changes in the unit cell parameters of zeolites.^105−107^ With the aid of operando synchrotron-based XRD experiments, we followed the structural changes of catalyst during the MTH reaction over HZSM-5, Ca/ZSM-5, and Na/ZSM-5 catalysts and explored correlations with the amount of retained adsorbates.

**Figure 12:**
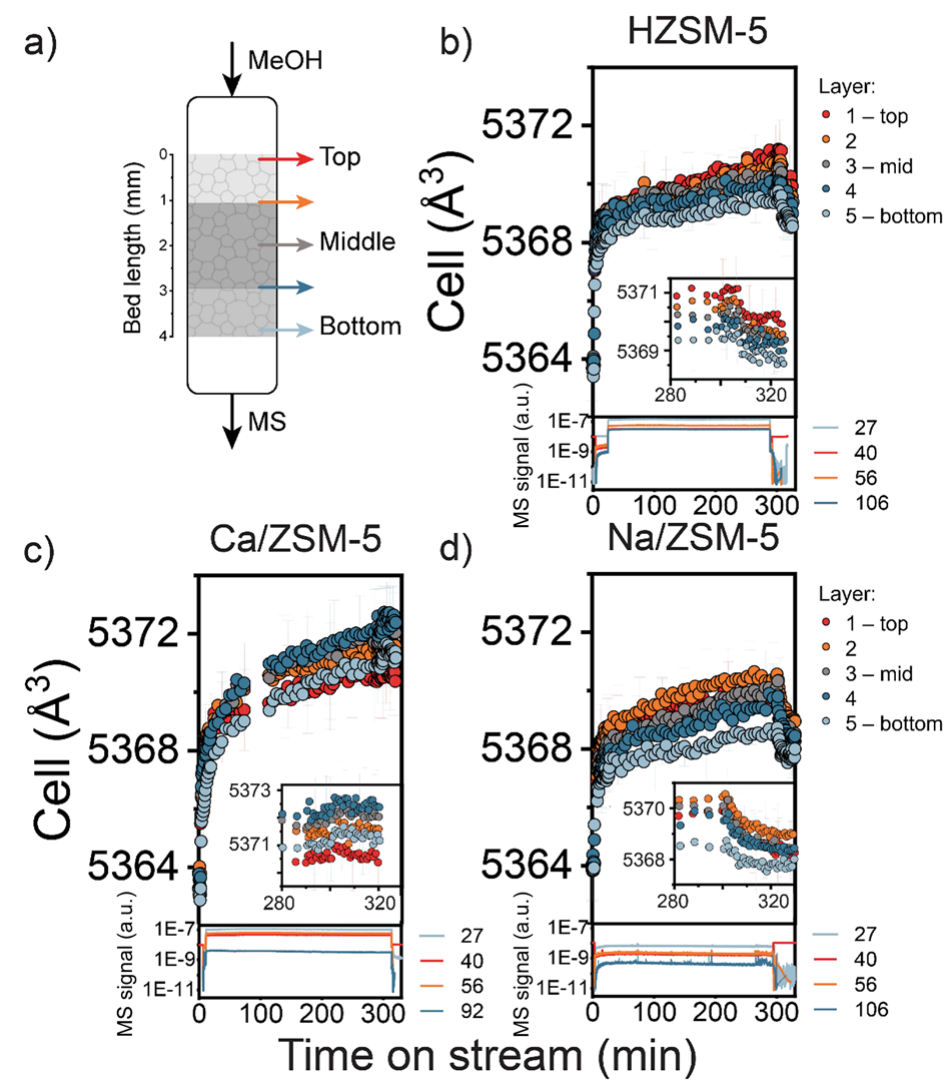
(a) Positions within the catalyst bed where the XRD data were acquired. (b–d) Unit cell volumes derived from Rietveld refinement of operando XRD data for HZSM-5, Ca/ZSM-5, and Na/ZSM-5 catalysts and after 5 h on stream and subsequent switch off the methanol for 30 min; MS spectra of the reaction are attached below. Conditions: 400 °C, 20 mg of catalyst, carrier −50 mL^–1^·min He, 13 kPa of MeOH.

For operando XRD measurements, a home-built Clausen-type flow cell^27^ placed on a movable sample stage was used. First, we acquired diffractograms of working catalysts at 5 different positions along the bed (Figures 12a and S25). Rietveld refinement was used to determine the unit cell parameters of the zeolite, which can be linked to the presence of hydrocarbons and other adsorbates such as oxygenates and water inside the pores.^105,108−110^Figure 12 shows the evolution of the unit cell volume together with MS data of the reaction cell effluent. The MS signals related to hydrocarbons (*m*/*z* = 27, 56, 92, and 106) point to the buildup of the hydrocarbon pool and the formation of common MTH products. The XRD results are generally in line with the TGA-MS results. Over HZSM-5 and Na/ZSM-5 catalysts, we observed a fast expansion of the unit cell during the initial stage of the MTH reaction, followed by a monotonous slow growth of the cell volume when the reaction proceeds in the steady-state regime (Figure 12b,d). Rapid cell expansion during the induction period and further gradual increase of the unit cell over time on stream have also been demonstrated for ZSM-22 zeolite in MTH reaction.^111^ A similar effect of light adsorbates on the unit cell was also observed by Kalantzopoulos et al. for HZSM-5.^112^ After the methanol was switched off, we observed the contraction of the unit cell for HZSM-5 and Na/ZSM-5 catalysts. In contrast, Ca/ZSM-5 demonstrated different dynamics of the pore expansion and contraction, specifically a larger increase of the cell volume upon exposure to methanol, which indicates that more adsorbates were retained in the presence of Ca^2+^ ions (Figure 12c). Following the 5 measurements positions along the catalyst bed, we generally observed the faster expansion of the unit cell volume at the beginning of the catalyst bed. This finding indicates a faster formation of hydrocarbon pool species in the active zone of the catalyst, starting from the reactor inlet. Comparing the cell expansion of the middle layer after 5 h on stream, the difference in cell volumes was higher for Ca/ZSM-5 catalysts than for HZSM-5 and Na/ZSM-5 (+0.16% vs +0.10 and +0.08%, respectively). Also, we did not observe any cell contraction after replacing the methanol-containing flow by dry He, supporting the previous findings of stronger retention of immobile adsorbates over Ca-modified catalysts. Next, we calculated the difference between the *a* and *b* unit cell parameters, which was found to be a suitable descriptor of catalyst deactivation by polyaromatic coke species (Figure S26).^109^ We indeed observed a decrease of the (*a* – *b*) parameter for the studied catalysts with time on stream, pointing to the ongoing deactivation. However, as deactivation after 5 h is not complete, the decrease in the (*a* – *b*) parameter is only minor.

#### Ex Situ XRD and TG Analysis

Since complete deactivation of the catalysts at given reaction conditions takes much longer than 5 h on methanol stream, we studied the difference between fresh and spent zeolites using ex situ synchrotron XRD^113^ together with TG analysis (Figures S27–S28). We observed that doublet peaks corresponding to *hkl* (0 5 1) and (−5 0 1/5 0 1) and *hkl* (0 10 0) and (10 0 0) reflections became single peaks for HZSM-5 and Na/ZSM-5 catalysts.^114^ This is the result of the *a* and *b* unit cell parameters becoming equal for fully deactivated HZSM-5 and Na/ZSM-5. Previously, such behavior has been associated with a deactivation mechanism that involves formation of rigid polyaromatic coke species inside the zeolite pores.^109,115^ In line with this, the (*a* – *b*) values for spent HZSM-5 and Na/ZSM-5 were close to zero (Table S4). At the same time, for a spent Ca/ZSM-5 the (0 5 1)/(−5 0 1/5 0 1) and (0 10 0)/(10 0 0) doublets at 1.60–1.65 Å^–1^ and 3.10–3.15 Å^–1^ are preserved. As a result, the (*a* – *b*) value is high for both fresh and spent Ca/ZSM-5 catalysts. The amount of coke and its combustion temperature derived from DTG curves were higher for HZSM-5 and Na/ZSM-5 (Figure S28 and Table S4). These findings suggest that deactivation of Ca/ZSM-5 proceeds through a different mechanism, likely involving lighter aromatic and aliphatic species in contrast to fused aromatic rings blocking the pores of HZSM-5 and Na/ZSM-5.

Altogether, XRD and TGA experiments supported the findings from ^13^C NMR and IR, namely that different amounts of intrazeolite adsorbates (hydrocarbons, water, and oxygenates) were observed in the pores of Ca/ZSM-5 as compared to the other two zeolites. We propose that strong adsorption of these molecules on Ca^2+^ sites changes the effective pore geometry, restricting the formation of aromatic hydrocarbon pool precursors and henceforth leading to the dominance of the olefin cycle.

## Conclusion

We compared the catalytic and structural properties of Ca/ZSM-5 and its reaction performance with those of HZSM-5 and Na/ZSM-5 catalysts. Using catalytic testing and PEPICO spectroscopy, we determined detailed product distributions over these catalysts. Ca/ZSM-5 demonstrated strongly suppressed hydrogen transfer and aromatization reactions, lower formaldehyde production, and promoted formation of C_3_–C_7_ olefins and paraffins. On the contrary, HZSM-5 and Na/ZSM-5 produced significant amounts of aromatics, including polymethylated benzenes and naphthalenes. Transient switching and TGA-MS experiments along with MAS NMR data show that Ca/ZSM-5 retains the highest amount of adsorbates, mainly represented by aliphatics, oxygenates, and water. The immobile, strongly adsorbed species, present in the pores during the reaction, occupy at least 10% of the total microporous volume. Operando IR showed that Ca/ZSM-5 preferentially retains water, methanol, and methoxy groups. Operando XRD analysis demonstrated that the formation of strong adsorbates leads to the highest expansion of Ca/ZSM-5 unit cell during methanol conversion and that this expansion cannot be reversed by removing methanol from the reaction feed. Ex situ XRD indicates that the deactivation mechanism of Ca/ZSM-5 is different from the formation of heavy polyaromatic species, as observed for HZSM-5 and Na/ZSM-5.

With this information, we link the higher catalytic stability and propylene selectivity of Ca/ZSM-5 to the larger amount of adsorbates (water, methanol, methoxy groups) retained in the pores of Ca/ZSM-5 during the MTH reaction. These immobile species occupy a significant fraction of the microporous volume and can inhibit the formation of bulky aromatic intermediates, shifting the reaction selectivity toward C_3_–C_7_ olefins and paraffins. These findings support the previous mechanistic studies of Gascon and co-workers, where suppressed growth of aromatic hydrocarbons over Ca/ZSM-5 was proposed to explain the increased propylene selectivity. Altogether, our findings illustrate how pore occupancy can influence the product distribution of the MTH reaction catalyzed by ZSM-5.

## Supporting Information Available

Catalyst preparation, methods for catalyst characterization, XRD patterns, ^1^H, ^27^Al, ^1^H – ^13^C SS MAS NMR results and discussion, SEM images, experimental design schemes, transient experiment results and their quantification, TGA results and discussion, IR results (PDF)
