# Supplementary material for: Ca Cations Impact the Local Environment inside HZSM-5 Pores during the Methanol-to-Hydrocarbons Reaction
Source: ACS Catal. 2023 Feb 23;13(6):3471–84. doi: 10.1021/acscatal.3c00059 (PMC10028611; doi:10.1021/acscatal.3c00059)
Supplement: Supplementary file 1 — cs3c00059_si_001.pdf [file cs3c00059_si_001.pdf]

Supporting information

to

Ca cations impact the local environment inside HZSM-5 pores during the methanol-to-hydrocarbons reaction

Anna Liutkova[a], Hao Zhang[a], Jérôme F.M. Simons[a], Brahim Mezari[a], Marta Mirolo[b], Gustavo A. Garcia[c], Emiel J.M. Hensen[a] *, Nikolay Kosinov[a] *

[a] Laboratory of Inorganic Materials and Catalysis, Department of Chemical Engineering and Chemistry, Eindhoven University of Technology, P.O. Box 513, 5600 MB Eindhoven, The Netherlands

[b] ESRF, The European Synchrotron, 71 Avenue des Martyrs, CS40220, 38043 Grenoble Cedex 9, France

[c] Synchrotron SOLEIL, L'Orme des Merisiers, St Aubin, B.P. 48, 91192 Gif sur Yvette, France

Corresponding authors:

Emiel J.M. Hensen

Tel: +31-40-2475178

E-mail: e.j.m.hensen@tue.nl

Nikolay Kosinov

Tel: +31-40-2478156

E-mail: n.a.kosinov@tue.nl

Table of Contents

Catalyst preparation 3

Catalyst characterization 3

Design of the operando experiments 7

Catalytic activity measurements: conversion and selectivity calculations 9

Transient GC-switching experiments 10

Traces of reaction components and contour maps 10

Quantification of GC-switches 13

TGA-MS results 15

MAS NMR of used catalysts 16

IR results 20

XRD analysis 23

References 26

Catalyst preparation

Four zeolite materials were used in the experiments: HZSM-5, Ca/ZSM-5, Na/ZSM-5, and Silicalite-1. The proton form of zeolite ZSM-5 was obtained by calcining a commercial NH_4ZSM-5 zeolite (Si/Al = 25, Alfa Aesar) at 550 °C for 5 h. HZSM-5 catalyst modified with calcium was prepared via incipient wetness impregnation of the calcined zeolite with aqueous solutions of $Ca(NO_3)_3$ (Alfa Aesar, 99.0%), following the procedure described elsewhere[1] aiming at 1 wt. % of metal loading. To obtain the partially ion-exchanged Na, a calculated amount of sodium nitrate (≥ 99% Merck) was dissolved in 100 mL demineralized water. The salt solution and 2 g of NH_4ZSM-5 were added to a round bottle ask, and the mixture was stirred at 60 °C for 3 h. After stirring, the mixture was transferred into a ThermoScientific Heraeus Megafuge 16 centrifuge. The liquid was removed by decantation and the solid was washed three times with demineralized water and centrifuged for 5 minutes (5000 rpm). The impregnated and exchanged samples were dried overnight at 110 °C and calcined at 550 °C for 5 h in static air. XRD patterns (Fig. S1) demonstrate that the original MFI topology is preserved in both samples. The relative intensity of Brønsted acid sites (BAS, signal at 4 ppm) was compared by ^{1}H MAS NMR analysis (Fig. S2). Morphology of ZSM-5 zeolite was preserved upon metal modification (Fig. S3). Amount of extraframework Al was estimated using ^{27}Al MAS NMR (Fig. S4). The preparation procedure and physico-chemical properties of a reference Silicalite-1 sample are provided elsewhere.[2]

Catalyst characterization

The elemental composition (Si/Al ratio and metal content) of the zeolite catalysts was determined by ICP-OES (Spectro CIROS CCD ICP optical emission spectrometer). The samples were dissolved in a 1:1:1 mixture of HF (40%), HNO_3 (65%) and H_2O prior to measurements.

The crystallinity of the zeolite samples was determined by powder X-ray diffraction (XRD). Ex situ XRD measurements were performed on a Bruker D2 powder diffraction system (Cu Kα radiation, scan speed 0.01°/s, 2θ range 5−60°) and ID15A beamline, ESRF. For the synchrotron experiments, photon wavelength was 0.0124 nm (100 keV). The incident beam was monochromated using a double-bounce bend Si(111) monochromator in the Laue geometry. The primary beam was focused to approximately 100 x 100 mkm^2 using a compound refractive lens transfocator. The detector distance and tilt were calibrated using NIST CeO_2 standard, and diffraction patterns were integrated using a locally modified version of the PyFAI package to eliminate outliers. Correction for detector transparency, homogeneity (flood field) and spatial distortion were applied at the time of integration. Further details can be found here.[3] Prior to measurements, sieved (250 – 500 μm) ZSM-5 catalyst (25 mg) was placed in a Kapton capillary (i.d. 1.83 mm, wall thickness 0.025 mm) sealed with wax. The integrated XRD patterns were analyzed by Rietveld refinement using the GSAS-II software. The patterns were refined in q-range of

0.4 – 7.5 Å^{-1}. The scale factor, background, and the unit cell parameters (P*nma* space group) were refined.

The morphology of the zeolite crystals was analyzed by scanning electron microscopy (SEM, FEI Quanta 200F scanning electron microscope at an accelerating voltage of 3 kV).

The acidic properties of zeolites were determined by IR spectroscopy of adsorbed pyridine as a probe molecule. Spectra were taken in the 4000 – 1000 cm^{-1} range using a Bruker Vertex 70v spectrometer. Samples were pressed into self-supporting wafers (10 − 20 mg, diameter 1.3 cm) and placed in an environmental cell. The wafers were pre-treated in O_2:N_2 (1:4 vol. ratio) flow at 550 °C (heating rate 10 °C·min^{-1}) to remove contaminants followed by cooling to 150 °C under dynamic vacuum ($p < 10^{-5}$ mbar). Afterwards the samples were exposed to an excess of pyridine vapor until saturation. Then the process of pyridine desorption was followed in a temperature range of 150 – 450 °C under dynamic vacuum, before the measurement the sample was first cooled to 150 °C and an IR spectra was recorded. For the experiments of pyridine adsorption with water preadsorbed, the pre-treated wafers were subjected to water vapors for 10 – 30 min at 2 kPa of water in 130 mL He ·min^{-1} until saturation. After that, the cell was degassed under dynamic vacuum, and the samples were exposed to an excess of pyridine vapor until saturation. The recording of IR spectra was carried out as ascribed above. For the quantification of Brønsted and Lewis acid sites, integral molar extinction coefficients of 0.73 cm·mol^{-1} and 1.11 cm·mol^{-1} were used.[4]

The conventional thermogravimetric analysis (TGA) of spent catalysts was performed using a Mettler Toledo TGA/DSC 1 instrument. An amount of used catalyst (≈ 10 mg) was placed in an alumina crucible and then heated up to 800 °C with a ramp rate of 5 °C·min^{-1} in O_2·He (20:40) 60 mL·min^{-1} flow.

Textural and adsorptive properties of zeolites were studied by Ar porosimetry at -186 °C using a Micromeritics ASAP2020 machine. Prior to measurements, the samples were pre-treated at 400 °C under evacuation. The microporous volume was calculated by the *t*-plot method using a thickness range from 3.5 to 4.5 Å.

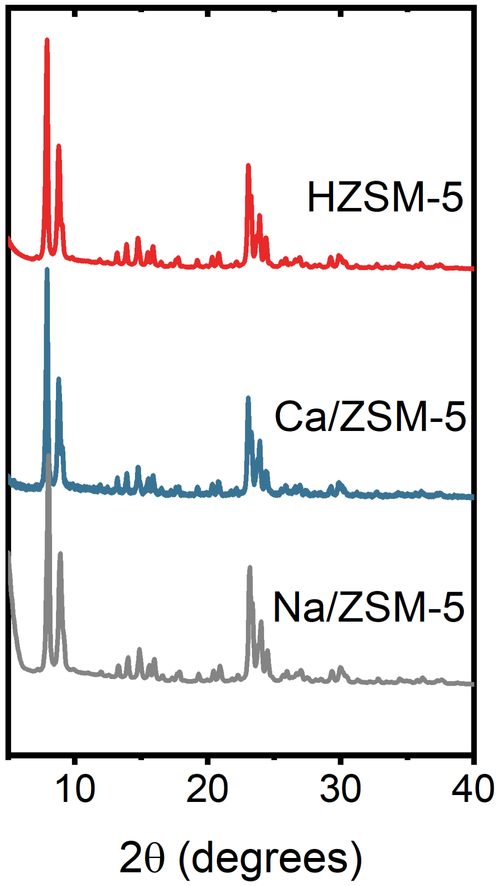

Figure S1. Lab-based XRD patterns of the prepared catalysts.

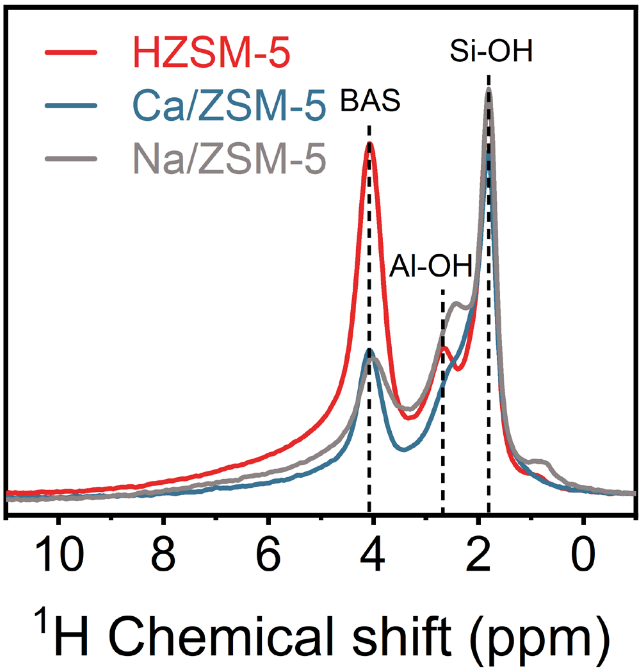

Figure S2. ^{1}H MAS NMR spectra of the prepared catalysts. The peak with a chemical shift of 4 ppm corresponds to isolated and H-bonded bridged Si-O(H)-Al respectively. The signals at 2.6 ppm and 1.8 ppm assigned to extra-framework Al-OH and Si-OH groups, respectively.

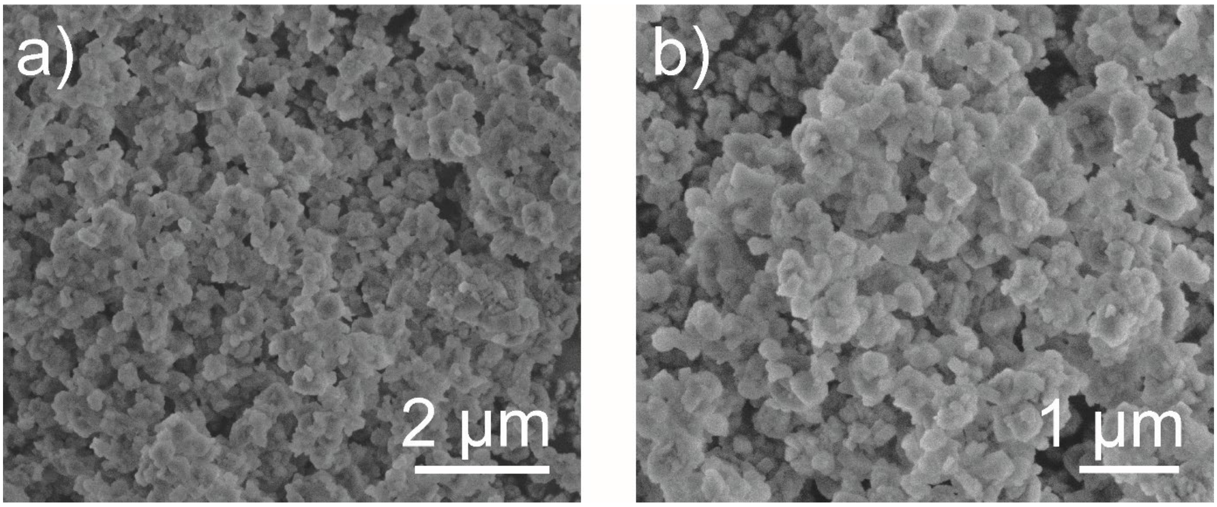

Figure S3. SEM images of (a) HZSM-5 and (b) Ca/ZSM-5 used in this study.

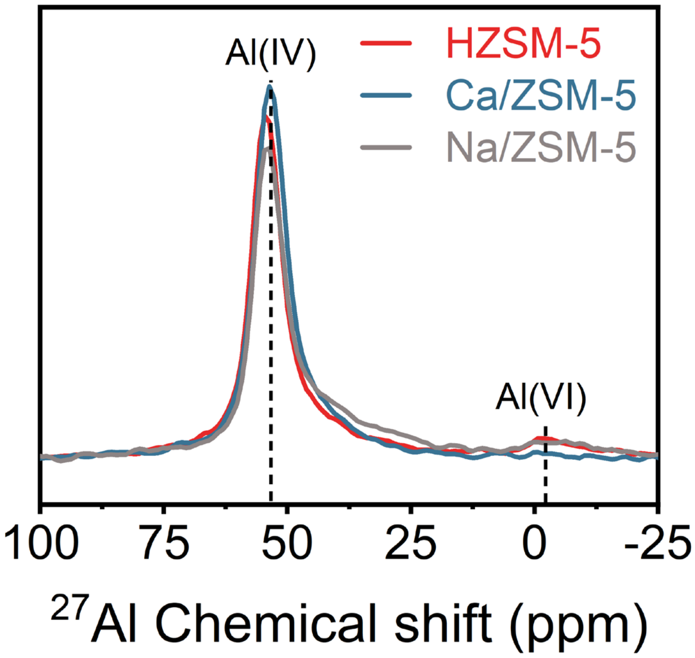

Figure S4. ^{27}Al MAS NMR spectra of the prepared catalysts. The peaks with a chemical shift of 53 ppm and 0 ppm correspond to tetrahedral framework Al (IV) and octahedral extra-framework Al (VI), respectively.

Design of the operando experiments

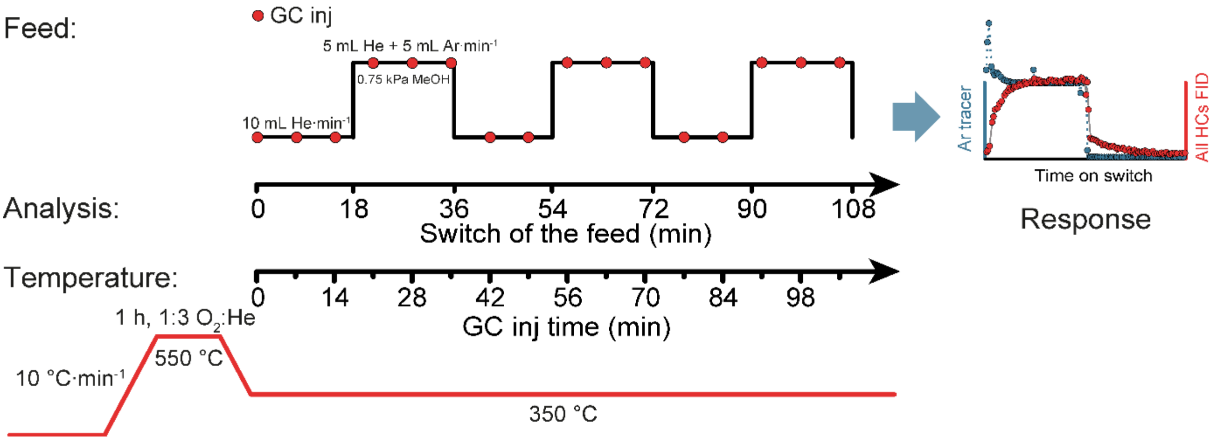

Figure S5. Design of the transient switching experiment.

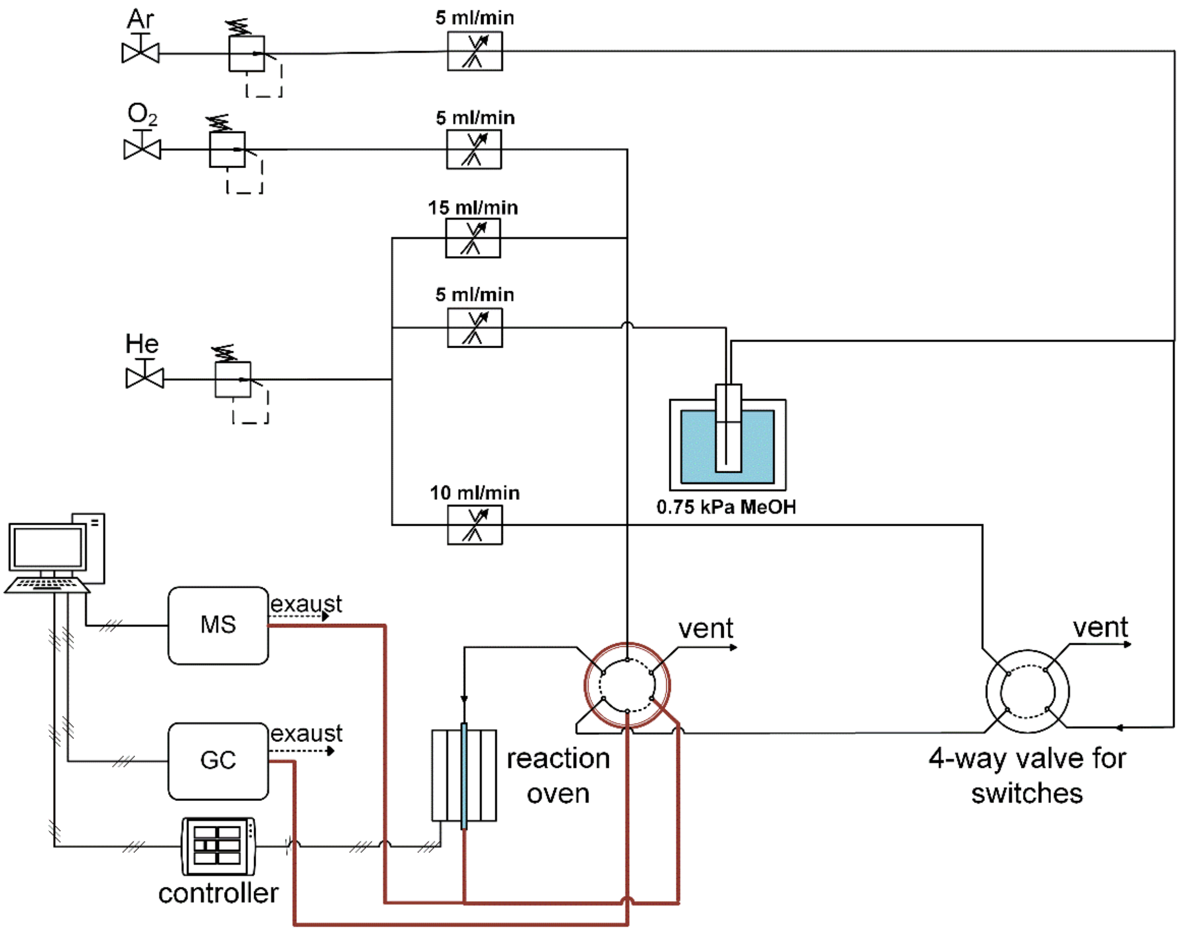

Figure S6. Principle scheme of the GC switching setup.

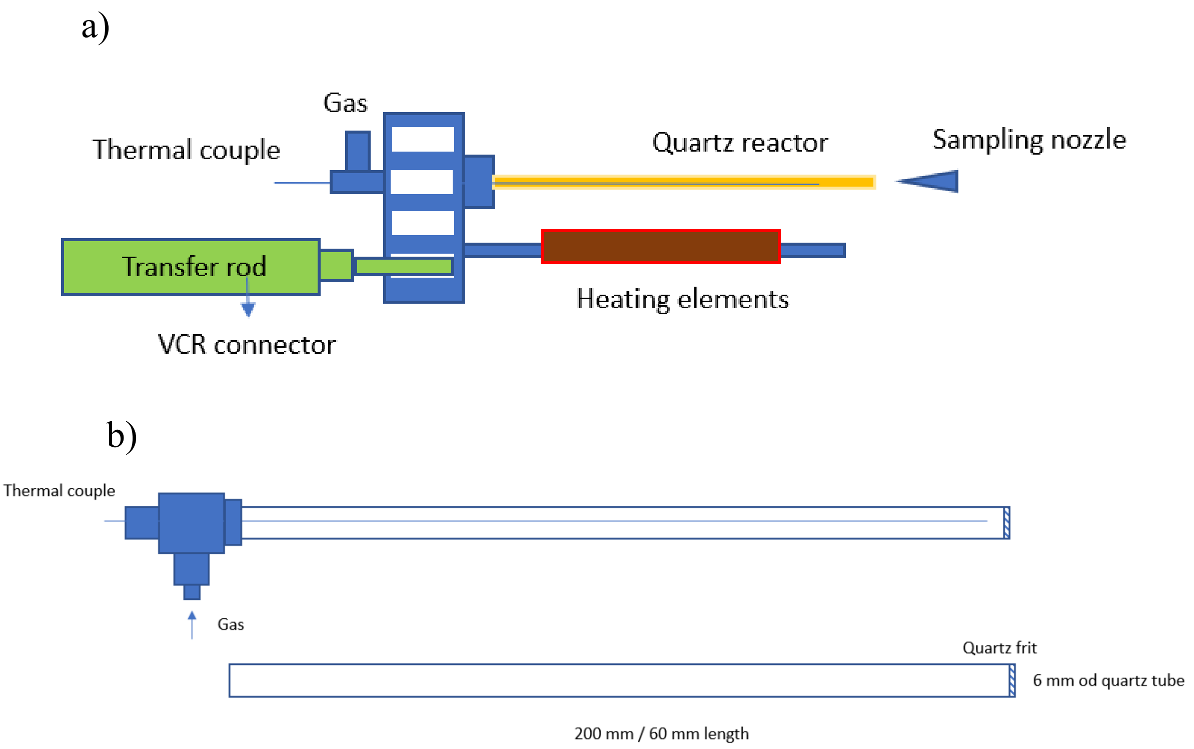

Figure S7. a) Reactor setup used for PEPICO measurements; b) quartz reactor.

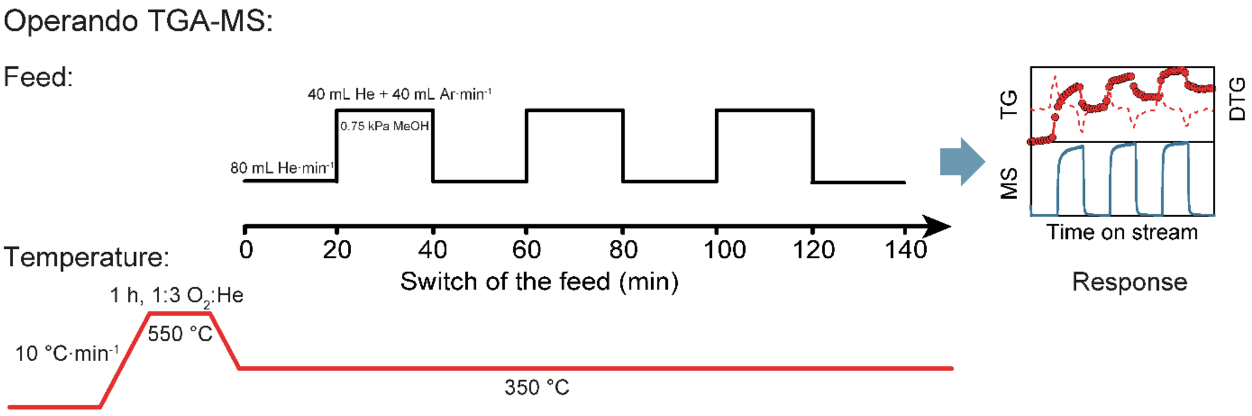

Figure S8. Design of the operando TGA-MS experiment.

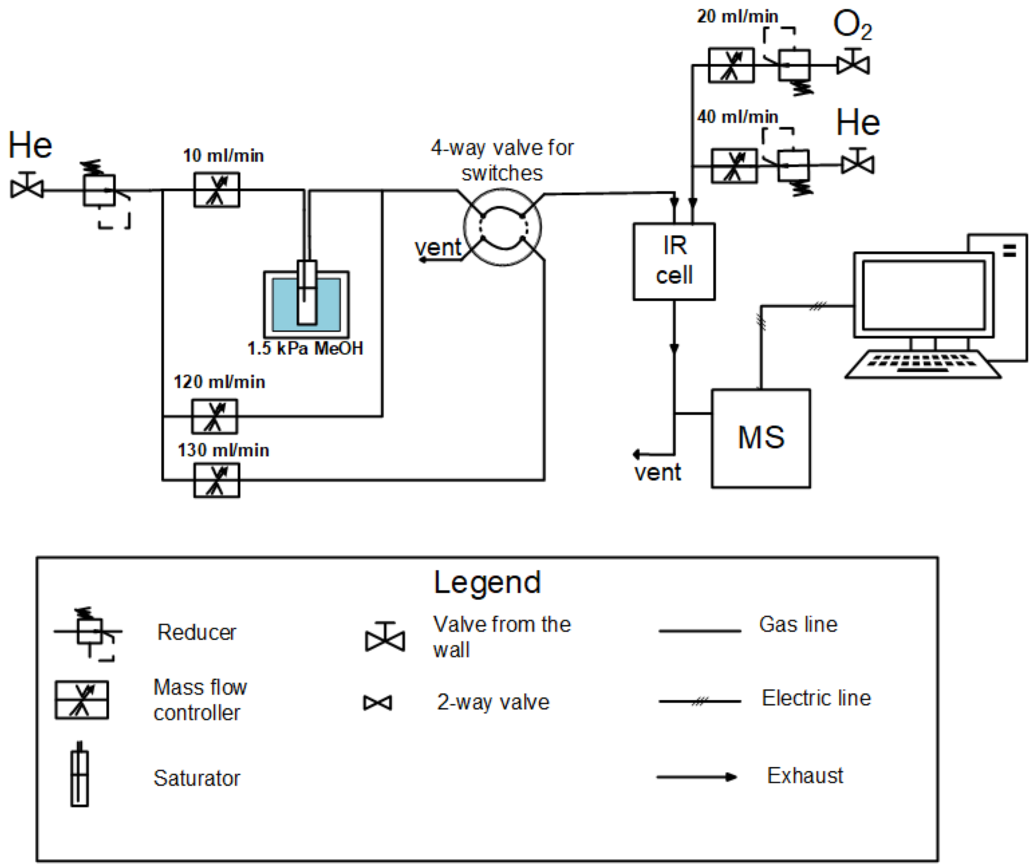

Figure S9. Principle scheme of the setup for operando IR switches.

Catalytic activity measurements: conversion and selectivity calculations

Performance of the catalysts was compared using throughput numbers and carbon-based selectivity. Methanol throughput is defined as the amount of methanol in g converted per g of catalyst before the conversion of light oxygenates drops below 75%. Selectivity of the catalysts obtained by the integration of each product/group of products of interest before the conversion of light oxygenates drops below 75%. For throughput calculations, conversion was defined as the carbon-based fraction of light oxygenates (methanol and dimethyl ether) consumed during the reaction:

$$X = \frac{n_{C,MeOH_{in}} - n_{C,MeOH_{out}} - 2 \times n_{C,DME_{out}}}{n_{C,MeOH_{in}}} \times 100\% \quad (1.1)$$

Further procedure was reproduced from here.[1,5]

Transient GC-switching experiments

Traces of reaction components and contour maps

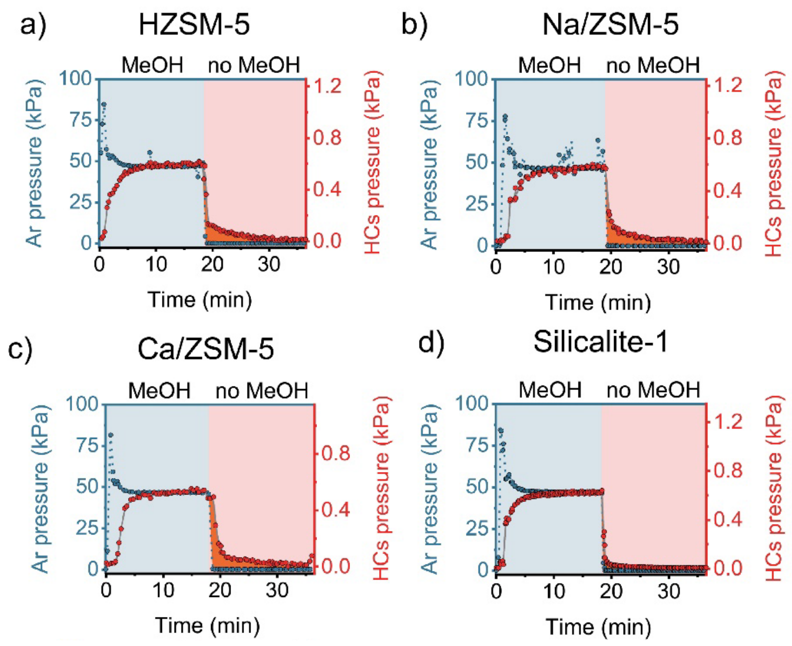

Figure S10. Step-response MTH experiments performed at 350 °C. Trace of integral amount of hydrocarbons being formed in presence and absence of methanol flow compared to Ar tracer are displayed. The area of interest – elution of hydrocarbons after the methanol was switched off is highlighted with orange. Conditions: 350 °C, 50 mg of catalyst, 18.3 min switch, 5 mL·min^{-1} He flow with 1.5 kPa of MeOH + 5 mL·min^{-1} side flow of Ar tracer.

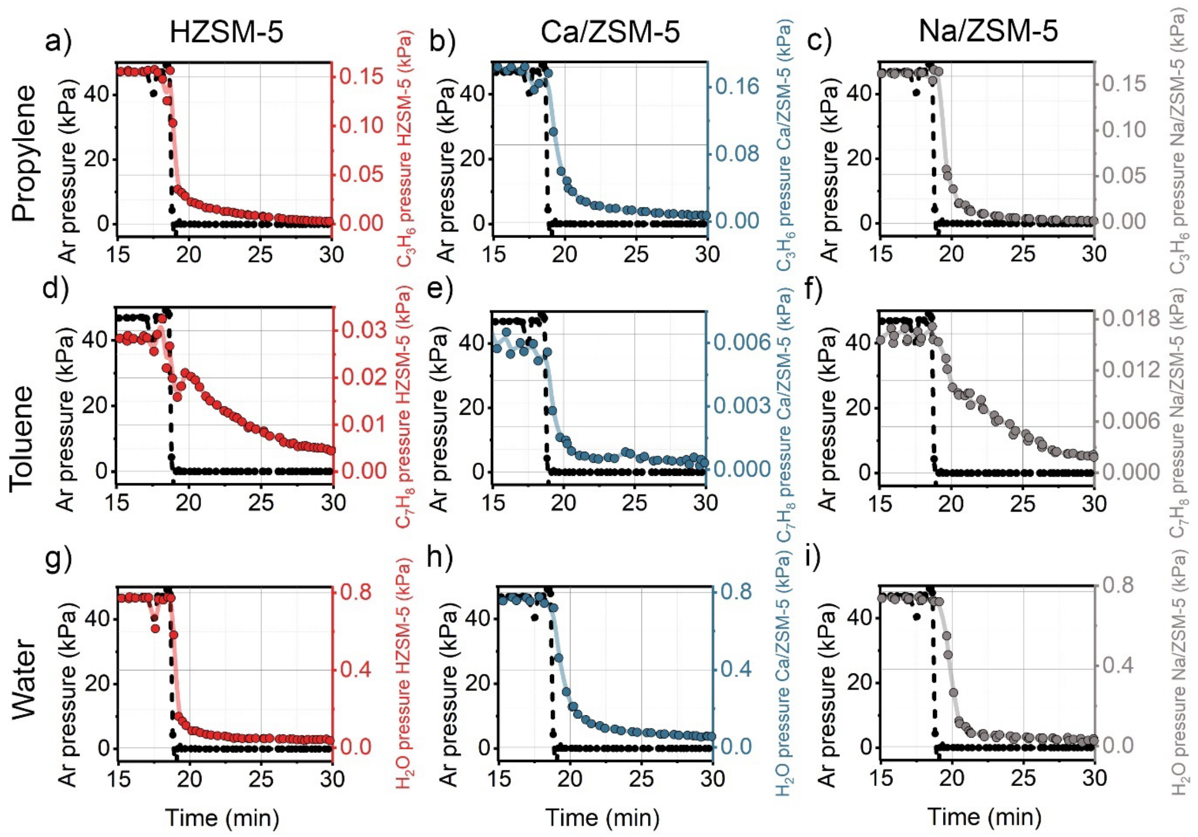

Figure S11. Step-response MTH experiments performed at 350 °C. Traces of individual products being formed in presence and absence of methanol flow compared to Ar tracer are displayed. Conditions: 350 °C; 50 mg of catalyst; 18.3 min switch, 5 mL·min^{-1} He flow with 1.5 kPa of MeOH + 5 mL·min^{-1} side flow of Ar tracer.

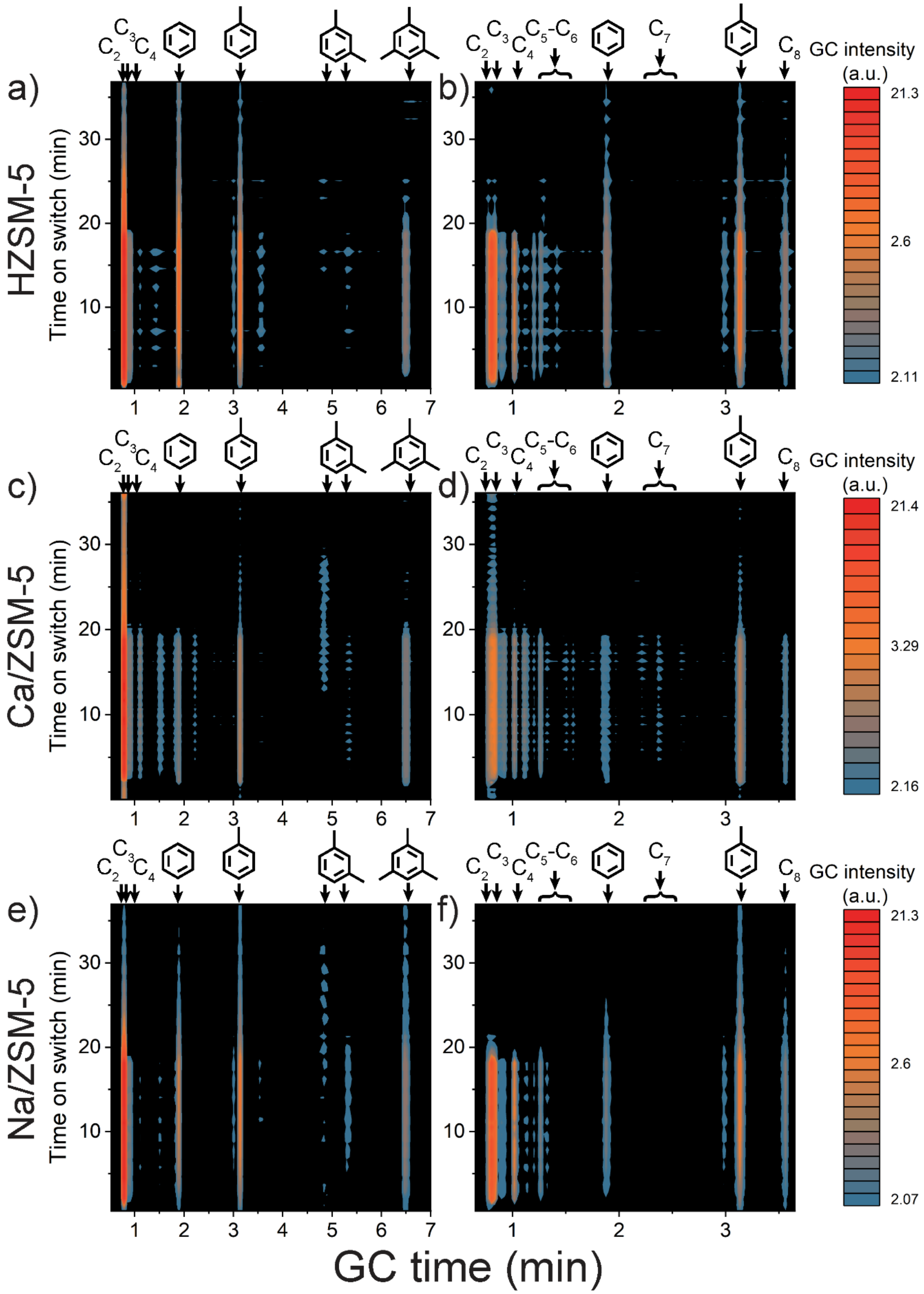

Figure S12. Contour maps from step-response MTH experiments obtained from chromatograms array and arranged according to time on switch: a,c,e – whole range of hydrocarbons observed from FID channel, b,d,f – enlarged region with light aliphatics and aromatics. Conditions: 350 °C, 50 mg of catalyst, 18.3 min switch, 5 mL·min^{-1} He flow with 1.5 kPa of MeOH + 5 mL·min^{-1} side flow of Ar tracer.

Quantification of GC-switches

To evaluate the amounts of water and hydrocarbons retained and eluted from the working catalysts (Fig. 5b), we quantified these amounts with the steps below:

First, we estimated theoretical amount of methanol and water, assuming 100% conversion of substrate and reaction ongoing according to the following scheme:

$$2MeOH \rightarrow H_2O + DME \rightarrow \underset{\text{HC pool}}{-(CH_2)-} + H_2O \quad (1.2)$$

We estimated that F_{MeOH} = 0.112 mg·min^{-1} assuming that P_{MeOH} = 0.75 kPa and total flow is 10 mL·min^{-1} (5 mL He·min^{-1} of saturator flow and 5 mL Ar·min^{-1} side flow). If time of the switch when feeding methanol is 18.3 min, then we convert 2.1 mg of methanol per one switch. This corresponds to 0.065 mmol of MeOH or 0.065 mmol of water produced.

Then we integrated overall amount of water and all hydrocarbons observed from FID channel (exemplified in Fig. S13) and converted it to mmol of water and carbon according to the following equation:

$$n_i = \frac{(t_n - t_{n-1}) \cdot p_i \cdot F_{carrier}}{P \cdot 60 \cdot 22.4}, \quad (1.3)$$

Where t is time on steam, s; $F_{carrier}$ is total flow, 10 mL·min^{-1} (5 mL He·min^{-1} of saturator flow and 5 mL Ar·min^{-1} side flow) for the given case; p_i is partial pressure of product of interest or methanol; 22.4 corresponds to the volume of 1 mole of ideal gas at STP, L·mol^{-1}; P – pressure in the system, 101.325 kPa.

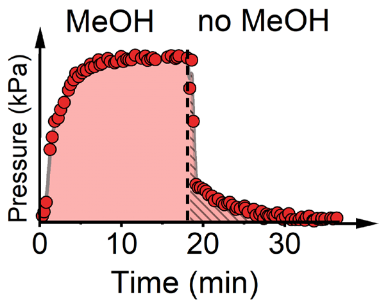

Figure S13. The GC-switching profile of all HC integrated from FID channel: red area – sum of all HCs produced over one switch, hatched area – amount of HCs eluted when methanol was switched off.

We estimated the uncertainties for GC-switching experiments and compared them to theoretical numbers (Table S1). While oxygen balance is close to 100% for experiments of the catalyst, a certain amount of hydrocarbons appears to be irreversibly retained during each switch. We should note here that procedure for calculating oxygen balance is accurate because it mainly depends on the relative sensitivity factor of water. The quantification of hydrocarbons is much more complex as it involves

many different molecules and varying sensitivity factors can lead to the lower overall calculation accuracy. We calibrated the GC for CH_4, C_2H_4, C_2H_6, C_3H_6, C_3H_8, benzene, and toluene and used carbon numbers to estimate the relative sensitivity factors of other molecules. From these measurements we found that average numbers from integration are 0.0045 ± 0.0003 mmol for HCs and 0.0068 ± 0.0001 mmol for water for HZSM-5 catalyst (Fig. S14, Table S1).

Table S1. Uncertainty estimations for GC switching experiments over HZSM-5 catalyst.

Comment	Exp1	Exp2	Exp3	Units
Amount of eluted hydrocarbons after switch	0.0048	0.0048	0.0040	mmol
Amount of eluted water after switch	0.0066	0.0070	0.0067	mmol

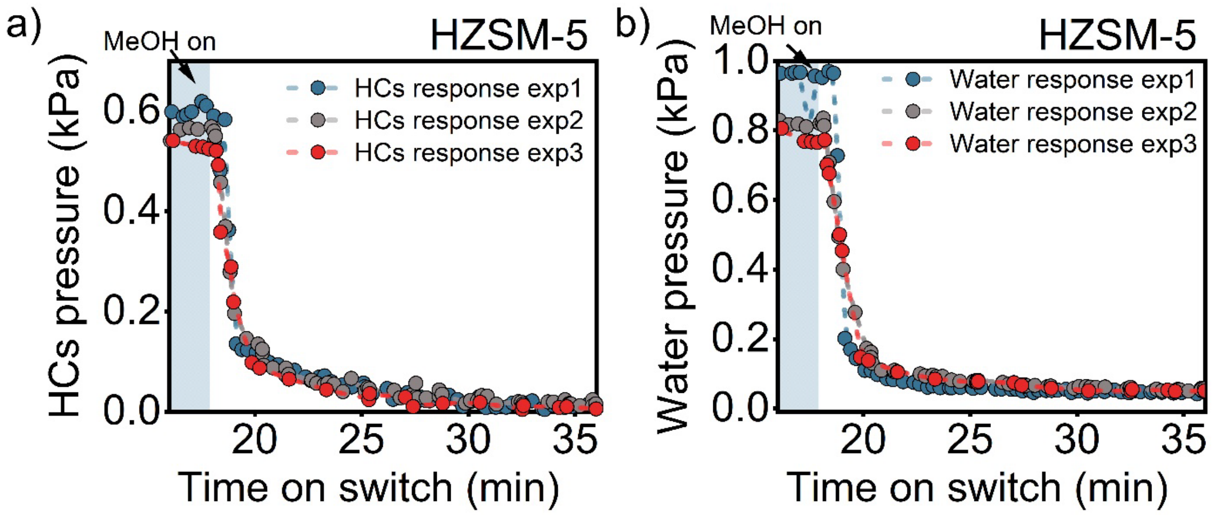

Figure S14. Three independent step-response MTH experiments performed at 350 °C: total hydrocarbon(a) and water (b) traces. Conditions: 350 °C, 50 mg of catalyst, 18.3 min switch, 5 mL·min^{-1} He flow with 1.5 kPa of MeOH + 5 mL·min^{-1} side flow of Ar tracer.

TGA-MS results

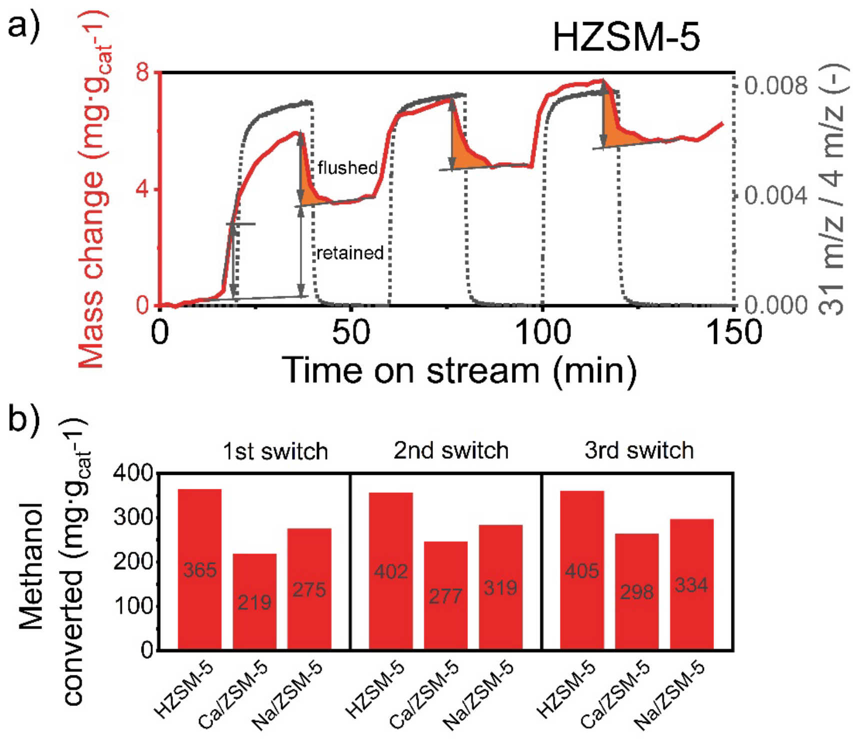

Figure S15. a) Example of TGA-MS data acquisition – TGA profile recorded following the change of adsorbate mass and MS profile of *m/z* 31 signal corresponding to methanol and normalized by *m/z* = 4 signal corresponding to He; b) converted methanol estimated per each switch from MS data. The amount of converted methanol was estimated from the comparison of *m/z* = 31 peak areas obtained in the respective experiments with the value obtained in a blank experiment using non-active Silicalite-1 material.

We suggest that presence of adsorbed species over Ca^{2+} centers hinders accessibility of reacting species to the acid sites. In order to check it, we carried out additional catalytic experiments with different Ca loading of parent ZSM-5. In addition to HZSM-5 and Ca/ZSM-5 (1wtCa/ZSM-5 below), we evaluated the performance of 2 more catalysts: 0.5wtCa/ZSM-5 and 1.5wtCa/ZSM-5, prepared using incipient wetness impregnation of HZSM-5. Properties of the Ca-modified catalysts are provided in Table S2. Kinetic experiments demonstrate that overall the catalyst stability of 0.5wtCa/ZSM-5 and 1wtCa/ZSM-5 were significantly higher than that for HZSM-5 (Fig. S16a). The sample with the highest loading (i.e., more than 1 Ca^{2+} per 2 Al sites) shows a strong decline in methanol conversion in both kinetic and TGA-MS experiments. To quantify the amount of adsorbates per Ca^{2+} metal, we carried out TGA-MS experiments over these samples (Fig. S16b). We estimated that the amount of retained adsorbates is 2 molecules per mol of Ca.

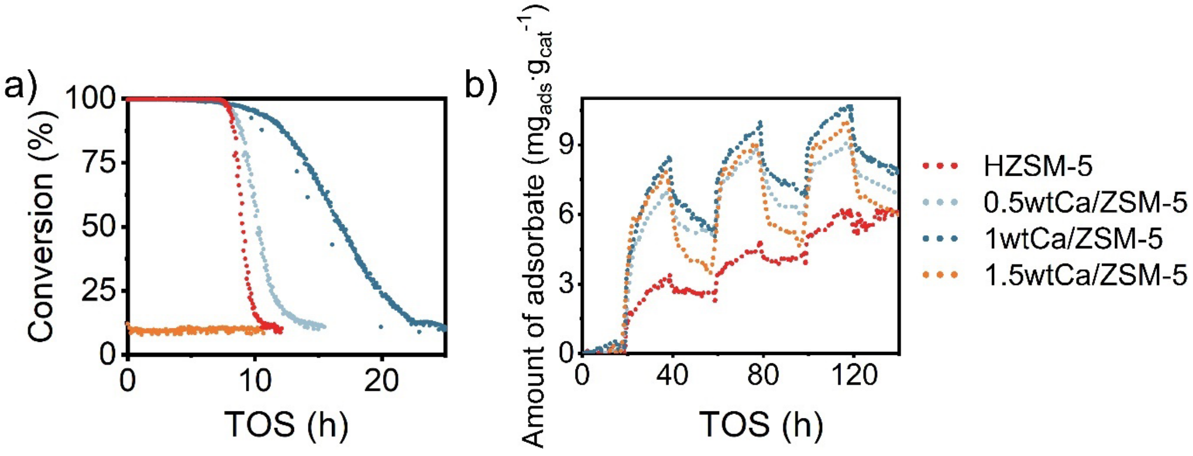

Figure S16. a) Conversion of methanol as function of time on stream for different Ca loadings. Reaction conditions: 450 °C, 25 mg of catalyst, 12.3 kPa of MeOH, carrier – 30 mL·min^{-1} He, WHSV 12 h^{-1}. b) TGA-MS profiles recorded during switching from methanol-containing He flow to dry He over HZSM-5 and ZSM-5 catalysts with different Ca loading. Conditions: 450 °C, 10 mg of catalyst, carrier – 80 mL·min^{-1} He, 0.75 kPa of MeOH.

Table S2. Properties of ZSM-5 catalysts, modified with different amount of Ca.

**Sample**	**%wt of Ca** [a]	**Ca/Al ratio, mol·mol^{-1}** [a]	**Ca content, mmol·g_{cat}^{-1}** [a]	**BAS/LAS, µmol·g^{-1}** [b]
HZSM-5	n.a.	n.a.	n.a.	437/85
0.5wtCa/ZSM-5	0.71	0.25	0.18	293/282
Ca/ZSM-5 (1wtCa/ZSM-5)	1.11	0.54	0.27	189/361
1.5wtCa/ZSM-5	1.56	0.71	0.39	120/311

a) Measured by ICP elemental analysis; b) IR spectroscopy of adsorbed pyridine.

MAS NMR of used catalysts

We performed ^{1}H-^{13}C NMR analysis of the used catalysts to study the hydrocarbon molecules occluded inside the catalyst pores during the MTH reaction. The samples were taken after the continuous flow experiments for 10 min TOS and 3 h TOS with ^{13}C labeled methanol to obtain higher signal intensity of the retained hydrocarbon species and the collected spectra are weight normalized. Different types of pulse sequences were implemented: CP MAS NMR which is sensitive to strongly adsorbed species, Hahn Echo MAS NMR to observe the mobile species, and ^{1}H – $^{13}C\{^{1}H\}$ HETCOR MAS NMR 2D sequence to correlate signals from the protons and carbons simultaneously. Two main regions for ^{1}H-^{13}C NMR spectra of HZSM-5 and Na/ZSM-5 are distinguished: 10 – 30 ppm, corresponding to aliphatic sp^{3} carbon atoms; and 120 – 150 ppm corresponding to sp^{2} aromatic and olefin carbon atoms

(Figs. S17 – S18).[5] Used HZSM-5 sample demonstrate typical ^{13}C NMR spectra of retained alkylated aromatic species (characteristic peaks at 134, 131, 128, 22 and 18 ppm).[6] In contrast, the spectrum of used Ca/ZSM-5 is completely different, featuring sharp signals at 49 and 59 ppm, which can be assigned to adsorbed DME and methanol molecules as well as methoxy species.[7,8] More detailed assignment of chemical shifts is provided in Table 2 in the main text. As the Hahn Echo and HPDec experiments are quantitative, we can estimate relative ratio of hydrocarbon species to oxygenates by integration of following areas. The results are provided in Table S3. After 3 h on stream we can observe that oxygenate contribution increased for Ca/ZSM-5 catalyst compared to 10 min TOS, while the aromatics/olefins prevail for H and Na/ZSM-5 catalysts pointing out to formation and condensation of coke species (Figs. S17 – S18).

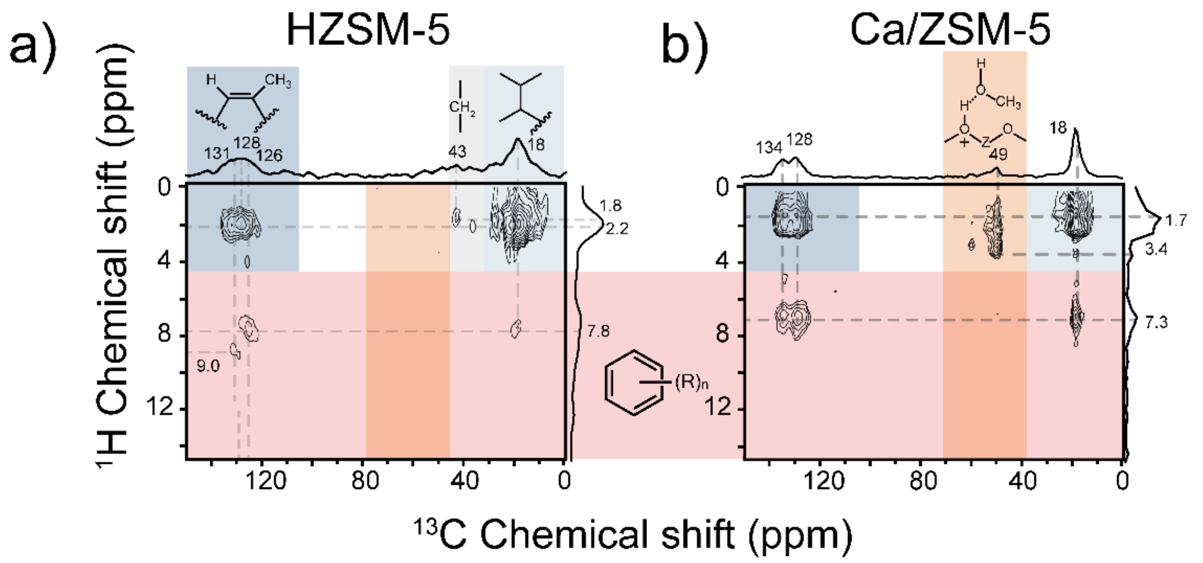

Figure S17. ^{1}H – ^{13}C{^{1}H} HETCOR MAS NMR 2D of the used catalysts from continuous flow experiments after 10 min of stream. Conditions: 350 °C, 100 mg of catalyst, carrier – 30 mL^{-1}·min He, 12 kPa of ^{13}C MeOH.

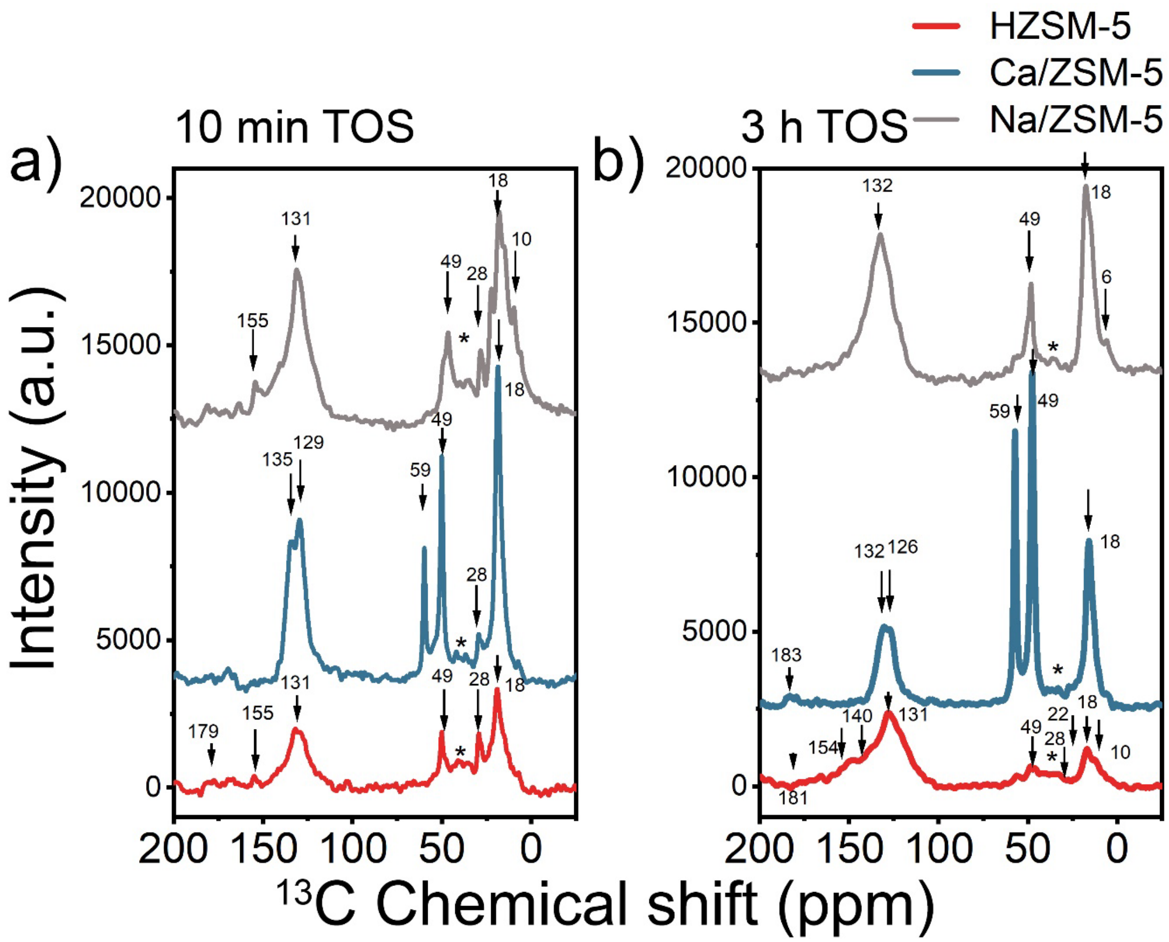

Figure S18. ^{1}H-^{13}C Hahn Echo MAS NMR of the used catalysts from continuous flow experiments after 10 min (a) and 3 h (b) on stream. Conditions: 350 °C, 100 mg of the catalyst, carrier – 30 mL^{-1}·min He, 12 kPa of ^{13}C MeOH. Sidebands are denoted with asterisks.

Table S3. Relative amount of aliphatics/aromatics and oxygenates estimated from direct excitation ^{1}H-^{13}C Hahn Echo MAS NMR and adsorbate content for the catalysts after different time on stream.

**Sample**	**Aliphatics + aromatics**		**Oxygenates**		**TGA weight loss**	
	**Area (a.u.)**	**(%)**	**Area (a.u.)**	**(%)**	**40 – 300 °C (% wt.)**	**300 – 800 °C (% wt.)**
10 min TOS						
HZSM-5	166521	84	31926	16	3.1	0.9
Ca/ZSM-5	326787	77	99599	23	2.6	1.5
Na/ZSM-5	545654	90	60246	10	2.2	1.5
3 h TOS						
HZSM-5	49129	91	4997	9	1.8	1.5
Ca/ZSM-5	45046	54	37896	46	2.5	1.4
Na/ZSM-5	26033	88	3656	12	2.1	1.1

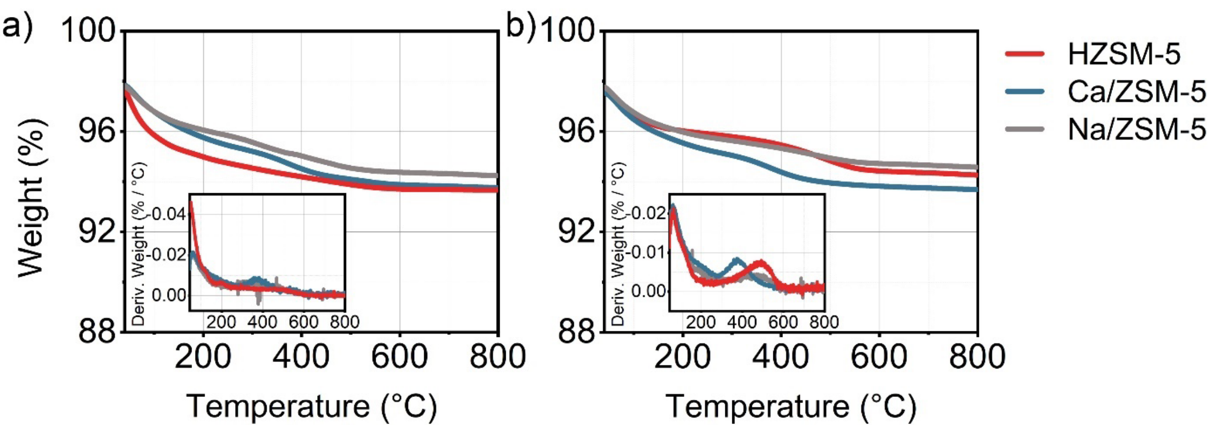

Figure S19. TG and DTG (inset) analysis for used samples after different TOS used for SS NMR: a) zeolite catalysts after 10 min on stream, b) after 3 h on stream. Used samples were obtained at the following conditions: 350 °C, 100 mg of catalyst, 12.3 kPa of ^{13}C MeOH, carrier – 30 $mL \cdot min^{-1}$ He.

IR results

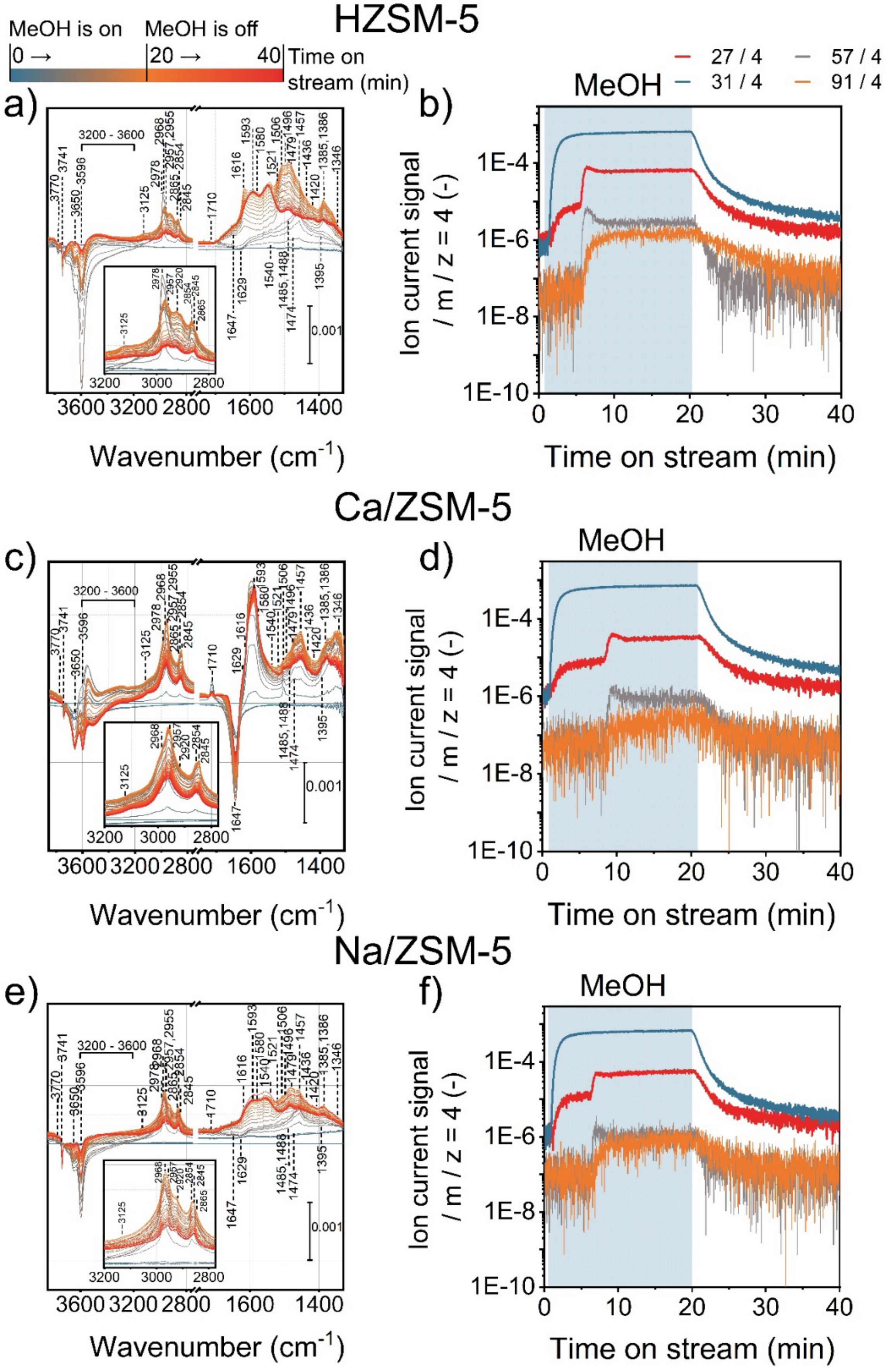
HZSM-5
MeOH is on
MeOH is off
0 →
20 →
40
Time on stream (min)
a)
b)
MeOH
27 / 4
31 / 4
57 / 4
91 / 4
Ion current signal / m / z = 4 (-)
Wavenumber (cm^{-1})
Time on stream (min)
Ca/ZSM-5
c)
d)
MeOH
Ion current signal / m / z = 4 (-)
Wavenumber (cm^{-1})
Time on stream (min)
Na/ZSM-5
e)
f)
MeOH
Ion current signal / m / z = 4 (-)
Wavenumber (cm^{-1})
Time on stream (min)

Figure S20. (a,c,e) Operando IR measurements combined with MS over ZSM-5 catalysts in presence and absence of the methanol feed and its corresponding Δ abs spectra: OH-region and hydrocarbon region. The Δabs spectra were obtained by subtraction of the first spectrum recorded at 350 °C in absence of methanol from all other spectra. (b,d,f) MS profiles normalized per *m/z* = 4 signal (He), signal *m/z* = 27 corresponds to ethylene, *m/z* = 31 – methanol, *m/z* = 57 – butane, and *m/z* = 91 to toluene. Conditions: 350 °C, 15 mg of catalyst pellet, carrier – 130 mL·min^{-1} He, 0.12 kPa of MeOH.

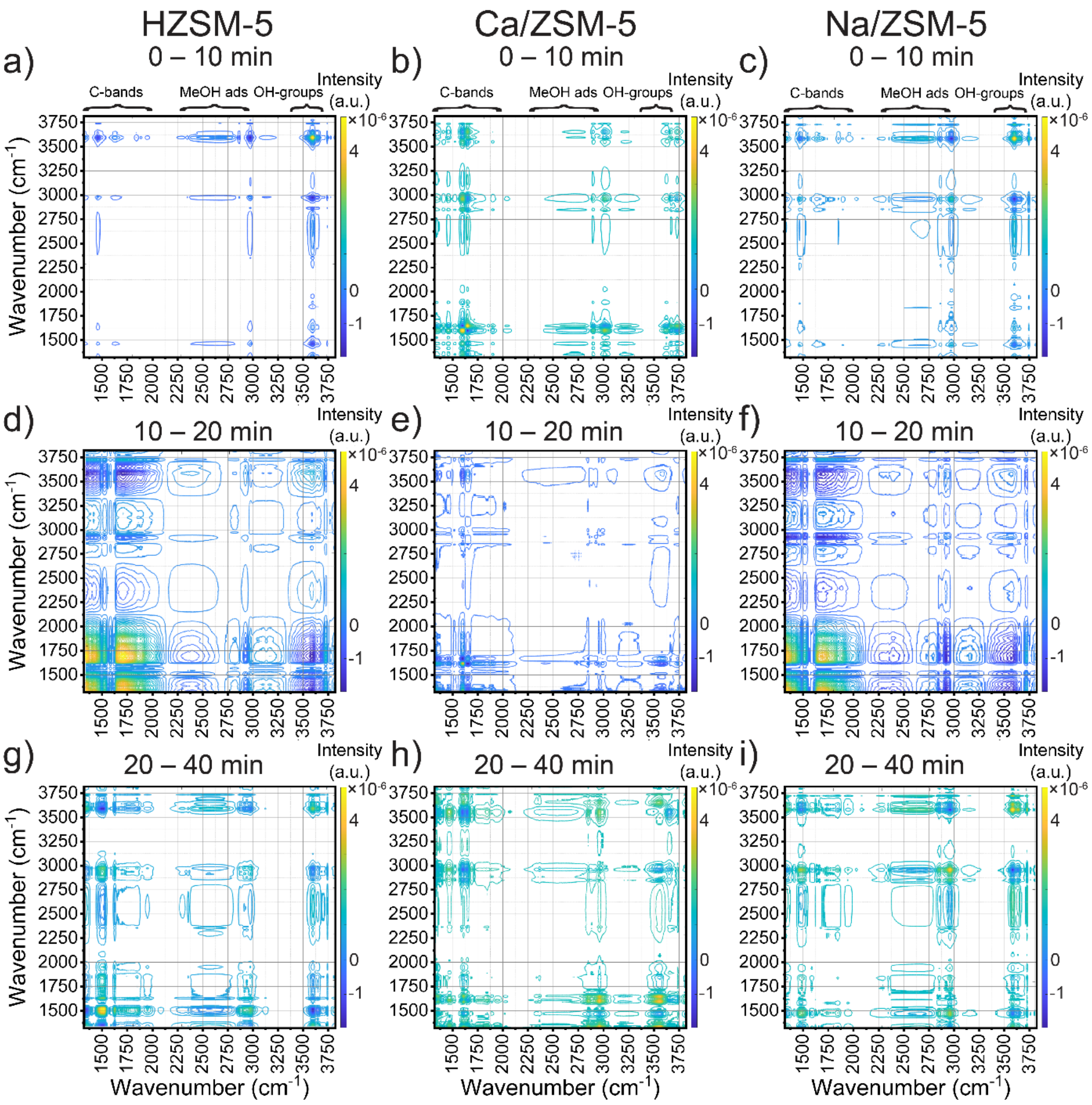

Figure S21. 2D correlation maps for IR measurements over ZSM-5 catalysts for different time on switch: (a – c) 10 min on switch, (d – f) 10 – 20 min on switch, in these two cases methanol is fed in the IR cell; (g – i) 20 – 40 min on switch when methanol feed is off. Conditions: 350 °C, 15 mg of catalyst pellet, carrier – 130 mL·min^{-1} He, 0.12 kPa of MeOH.

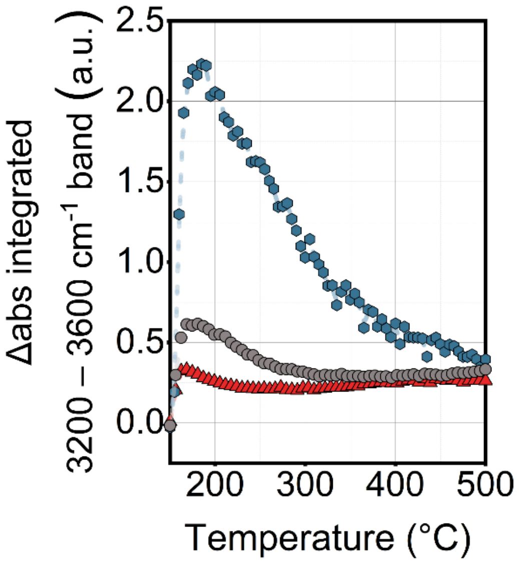

Figure S22. Area of interest integrated from Δ abs spectra from operando IR measurements for ZSM-5 catalysts during temperature-programmed experiments with water. Conditions: 150 – 500 °C, heating rate 5 °C·min^{-1}, 15 – 20 mg of catalysts, carrier – 130 mL·min^{-1} He, 0.07 kPa of water.

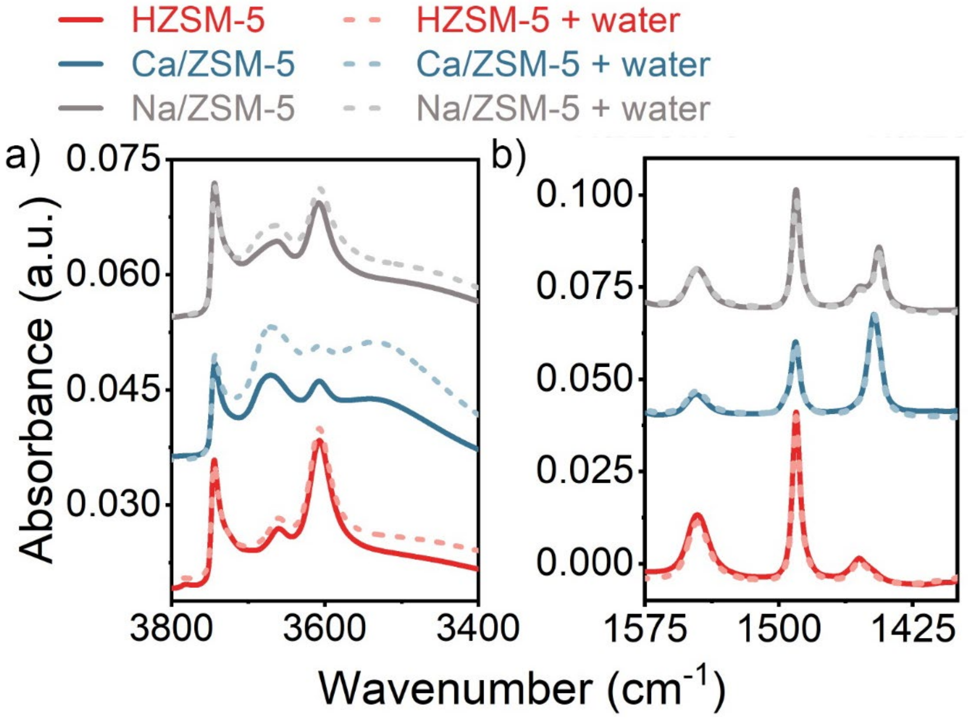

Figure S23. OH-stretching region before pyridine adsorption (a) and pyridine adsorption region (b) with pyridine adsorbed of three zeolite catalysts with (dashed) and without water (solid lines) preabsorbed.

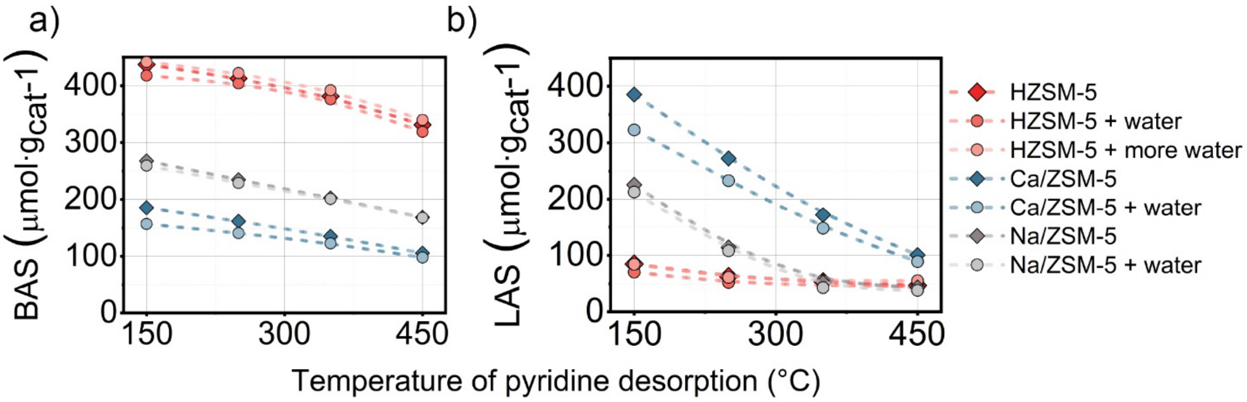

Figure S24. Amount of pyridine adsorbed over BAS and LAS of four zeolite catalysts with and without water preabsorbed.

XRD analysis

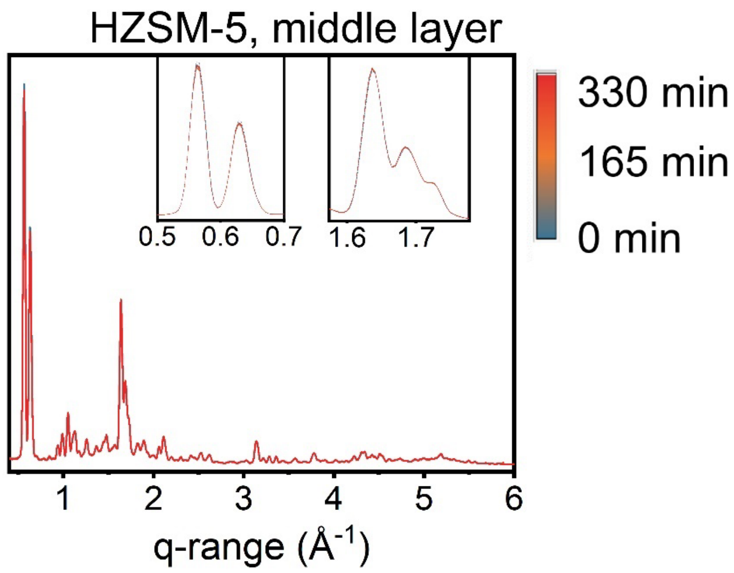

Figure S25. XRD patterns of HZSM-5 catalyst recorded in the middle of the catalyst during 5.5 h of an operando experiment with methanol was switched on at t = 10 min. Conditions: 400 °C, 20 mg of catalyst, carrier – 50 mL^{-1}·min He, 13 kPa of MeOH.

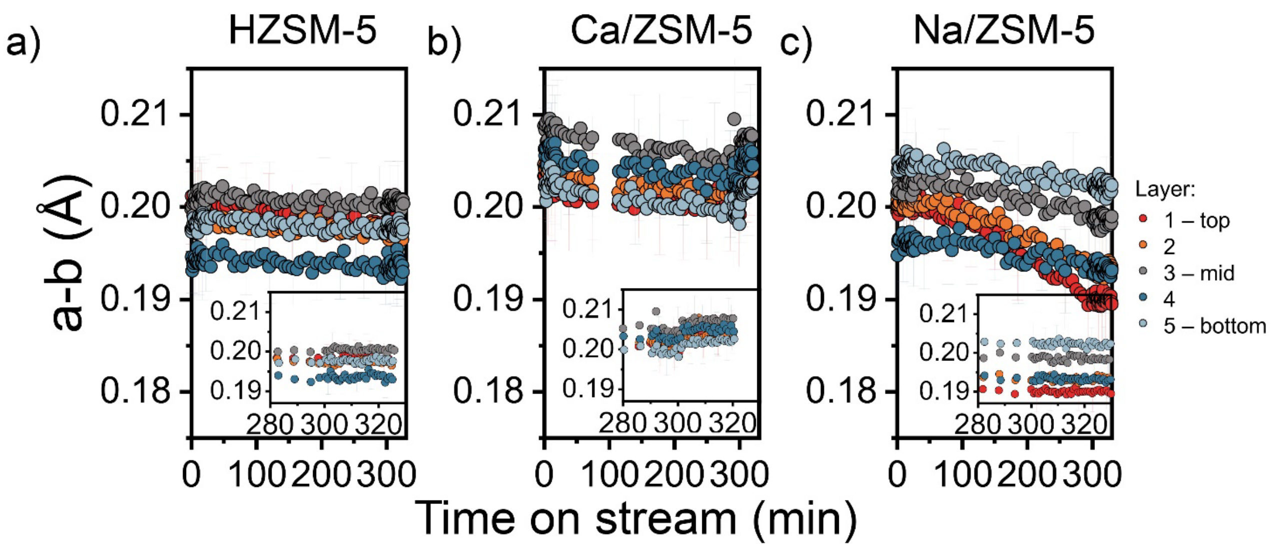

Figure S26. Difference of unit cell vectors a and b derived from Rietveld refinement of operando XRD data for HZSM-5, Ca/ZSM-5 and Na/ZSM-5 catalysts and after 5 h on stream and subsequent switch off the methanol for 30 min. Conditions: 400 °C, 20 mg of catalyst, 13 kPa of MeOH, carrier – 50 mL^{-1}·min He.

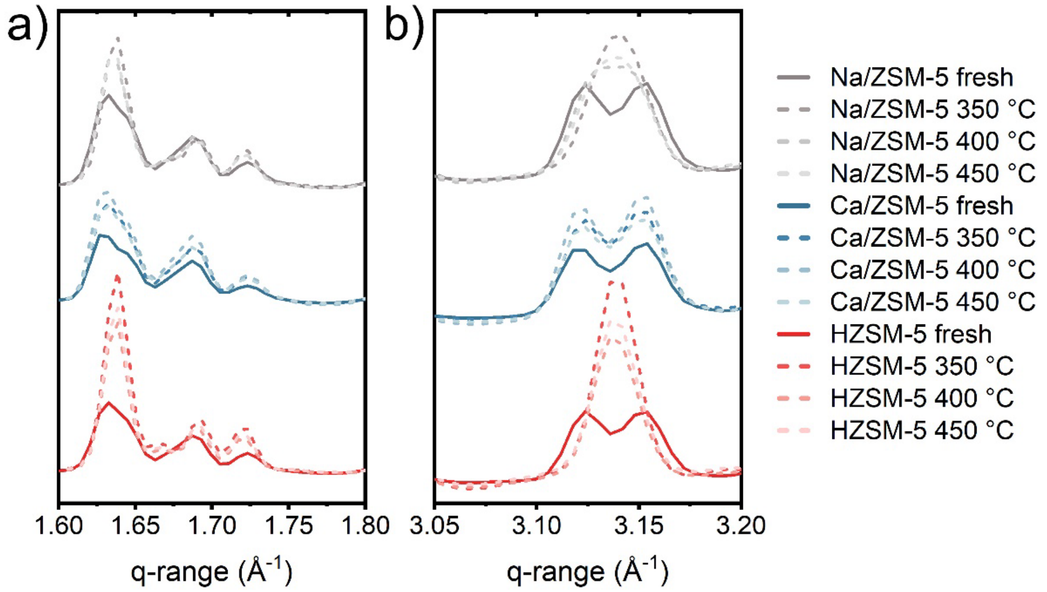

Figure S27. Zoom in of the X-ray diffractograms of the prepared (fresh) and spent (deactivated) catalysts at different temperatures. Deactivated samples were obtained at the following reaction conditions: 350 °C – 25 mg of catalyst, 0.75 kPa of MeOH, carrier – 10 mL·min^{-1} He, WHSV 0.3 h^{-1}; 400 °C – 25 mg of catalyst, 6 kPa of MeOH, carrier – 30 mL·min^{-1} He, WHSV 6 h^{-1}; 450 °C – 25 mg of catalyst, 12.3 kPa of MeOH, carrier – 30 mL·min^{-1} He, WHSV 12 h^{-1}. The regions correspond to hkl (0 5 1) and (-5 0 1/5 0 1) diffraction peaks (a) and hkl (0 10 0) and (10 0 0) (b) at wavelength = 0.0124 nm.[9]

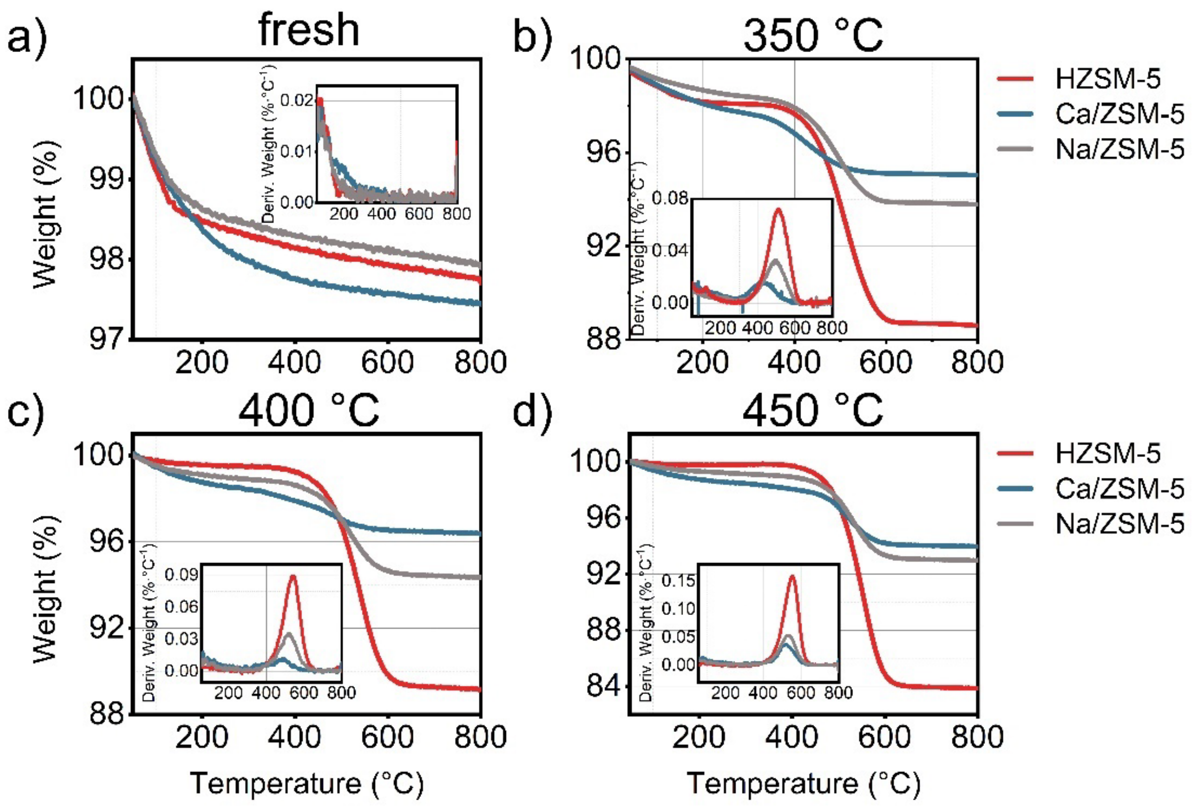

Figure S28. TG and DTG (inset) analysis of fresh and spent catalysts. Deactivated samples were obtained at the following reaction conditions: 350 °C – 25 mg of catalyst, 0.75 kPa of MeOH, carrier – 10 mL·min^{-1} He, WHSV 0.3 h^{-1}; 400 °C – 25 mg of catalyst, 6 kPa of MeOH, carrier – 30 mL·min^{-1} He, WHSV 6 h^{-1}; 450 °C – 25 mg of catalyst, 12.3 kPa of MeOH, carrier – 30 mL·min^{-1} He, WHSV 12 h^{-1}.

Table S4. Unit cell parameters and TG analysis for fresh and deactivated ZSM-5 catalysts.

Sample	*a*-unit cell (Å)	*b*-unit cell (Å)	(*a-b*) (Å)	Δ cell volume, fresh - deactivated (Å^3)	TGA weight loss		Maximum of DTG curve (°C)
					40 – 300 °C (% wt.)	300 – 800 °C (% wt.)	
HZSM-5 fresh	20.126	19.928	0.198	-	1.7	0.6	-
HZSM-5 deactivated at 350 °C	20.021	20.019	0.002	-2.0	1.4	9.5	510
HZSM-5 deactivated at 400 °C	20.034	20.022	0.012	6.0	0.5	10.3	543
HZSM-5 deactivated at 450 °C	20.034	20.023	0.011	4.1	0.2	15.9	554
Ca/ZSM-5 fresh	20.149	19.925	0.224	-	2.0	0.5	-
Ca/ZSM-5	20.138	19.936	0.202	1.4	1.2	4.6	430

deactivated at 350 °C							
Ca/ZSM-5 deactivated at 400 °C	20.151	19.935	0.216	5.3	1.6	2.1	485
Ca/ZSM-5 deactivated at 450 °C	20.144	19.938	0.206	4.2	1.5	4.5	514
Na/ZSM-5 fresh	20.131	19.927	0.203	-	1.6	0.5	-
Na/ZSM-5 deactivated at 350 °C	20.074	19.983	0.091	0.3	1.8	2.6	495
Na/ZSM-5 deactivated at 400 °C	20.103	19.971	0.132	6.8	1.1	4.6	518
Na/ZSM-5 deactivated at 450 °C	20.087	19.980	0.108	4.9	0.9	6.2	530

References

1. Yarulina, I. *et al.* Suppression of the Aromatic Cycle in Methanol-to-Olefins Reaction over ZSM-5 by Post-Synthetic Modification Using Calcium. *ChemCatChem* **8**, 3057–3063 (2016).

2. Liutkova, A. *et al.* A scanning pulse reaction technique for transient analysis of the methanol-to-hydrocarbons reaction. *Catal. Today* xxx (xxxx) xxx (2022) doi:https://doi.org/10.1016/j.cattod.2022.05.005.

3. Vaughan, G. B. M. *et al.* ID15A at the ESRF–a beamline for high speed operando X-ray diffraction, diffraction tomography and total scattering. *J. Synchrotron Radiat.* **27**, 515–528 (2020).

4. Datka, J., Turek, A. M., Jehng, J. M. & Wachs, I. E. Acidic properties of supported niobium oxide catalysts: An infrared spectroscopy investigation. *J. Catal.* **135**, 186–199 (1992).

5. Uslamin, E. A. *et al.* Co-Aromatization of Furan and Methanol over ZSM-5—A Pathway to Bio-Aromatics. *ACS Catal.* **9**, 8547–8554 (2019).

6. Epelde, E. *et al.* Differences among the deactivation pathway of HZSM-5 zeolite and SAPO-34 in the transformation of ethylene or 1-butene to propylene. *Microporous Mesoporous Mater.* **195**, 284–293 (2014).

7. Wang, W., Seiler, M. & Hunger, M. Role of Surface Methoxy Species in the Conversion of Methanol to Dimethyl Ether on Acidic Zeolites Investigated by in Situ Stopped-Flow MAS

NMR Spectroscopy. *J. Phys. Chem. B* **105**, 12553–12558 (2001).

8. Wang, W., Jiang, Y. & Hunger, M. Mechanistic investigations of the methanol-to-olefin (MTO) process on acidic zeolite catalysts by in situ solid-state NMR spectroscopy. *Catal. Today* **113**, 102–114 (2006).

9. Treacy, M. M. J. & Higgins, J. B. *Collection of simulated XRD powder patterns for zeolites fifth (5th) revised edition*. (Elsevier, 2007), MFI pattern, 278.
